# Efficient Defect-Driven Cation Exchange beyond the Nanoscale Semiconductors toward Antibacterial Functionalization

**DOI:** 10.1021/acsami.4c11425

**Published:** 2024-10-30

**Authors:** Svetlana Polivtseva, Olga Volobujeva, Ivan Kuznietsov, Reelika Kaupmees, Mati Danilson, Jüri Krustok, Palanivel Molaiyan, Tao Hu, Ulla Lassi, Mihhail Klopov, Heleen van Gog, Marijn A. van Huis, Harleen Kaur, Angela Ivask, Merilin Rosenberg, Nicholas Gathergood, Chaoying Ni, Maarja Grossberg-Kuusk

**Affiliations:** † School of Engineering, Department of Materials and Environmental Technology, TalTech, Ehitajate tee 5, 19086 Tallinn, Estonia; ‡ School of Science, Department of Cybernetics, TalTech, Ehitajate tee 5, 19086 Tallinn, Estonia; § Faculty of Technology, Research Unit of Sustainable Chemistry, University of Oulu, Pentti Kaiteran katu 1, 90014 Oulu, Finland; ∥ Nanostructured Materials and Interfaces, Zernike Institute for Advanced Materials, University of Groningen, Nijenborgh 4, 9747AG Groningen, The Netherlands; ⊥ Soft Condensed Matter, Debye Institute for Nanomaterials Science, Utrecht University, Princetonplein 5, 3584 CC Utrecht, The Netherlands; # Institute of Molecular and Cell Biology, University of Tartu, Riia 23, 51010 Tartu, Estonia; ∇ School of Chemistry, College of Science, University of Lincoln, Brayford Pool, Lincoln, Lincolnshire LN6 7TS, U.K.; ○ Department of Materials Science and Engineering, University of Delaware, Newark, Delaware 19716, United States

**Keywords:** thin films, defect chemistry, photoluminescence, materials design, ion exchange, doping V−VI metal chalcogenides, DFT calculation, antibacterial materials

## Abstract

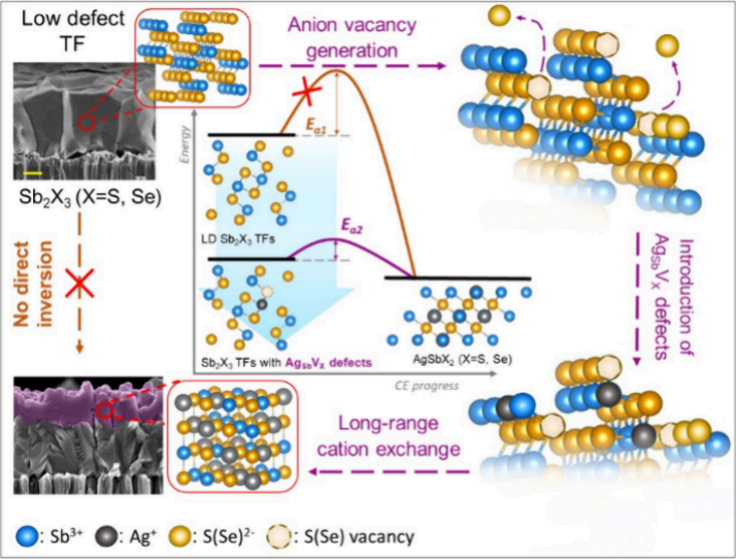

Defect engineering is an exciting tool for customizing semiconductors’ structural and optoelectronic properties. Elaborating programmable methodologies to circumvent energy constraints in multievent inversions expands our understanding of the mechanisms governing the functionalization of nanomaterials. Herein, we introduce a novel strategy based on defect incorporation and solution rationalization, which triggers energetically unfavorable cation exchange reactions in extended solids. Using Sb_2_X_3_ + Ag (I) → Ag: Sb_2_X_3_ (X= S, Se) as a system to model, we demonstrate that incorporating chalcogen vacancies and Ag_Sb_V_X_ complex defects into initial thin films (TFs) is crucial for activating long-range solid-state ion diffusion. Additional regulation of the Lewis acidity of auxiliary chemicals provides an exceptional conversion yield of the Ag precursor into a solid-state product up to 90%, simultaneously transforming upper matrix layers into AgSbX_2_. The proposed strategy enables tailoring radiative recombination processes, offers efficiency to invert TFs at moderate temperatures quickly, and yields structures of large areas with substantial antibacterial activity in visible light for a particular inversion system. Similar customization can be applied to most sulfides/selenides with controlled reaction yields.

## 1 Introduction

Precise structure and composition customization for semiconductor solids on substrates becomes crucial due to their perspectives in energy storage,^1^ catalysis,^2,3^ optronics,^4^ and biomedicine.^5,6^ Each technology is successful on an industrial scale when the quality of materials being developed is accompanied by an adequate conversion of reactants into desired products. Most state-of-the-art approaches for depositing TFs and tailoring their properties are characterized by low conversion efficiency of initial reactants into desired products.^7−10^ Such aspects create a bottleneck in essential chemical processes and require urgent adjustments, particularly in the current global economy fragmentation with technological decoupling and raw material chain failures.^11,12^

Although some reactions resulting in a solid matrix seem simple, a delicate balance between thermodynamics and kinetics regulates the process outcomes and the structure of products. Even though favored thermodynamics hints at the theoretical feasibility and spontaneity of an overall reaction,^13^ kinetic constraints may impede the desired organization of TFs on substrates with a reliable yield accuracy. Changing cation sites at the minimized rearrangement of anion frameworks may offer an unprecedented opportunity to address rational reactant consumption on a substrate while preserving materials’ structural and compositional diversity. Previous experimental and theoretical observations have validated that cation exchange can efficiently convert semiconductor II–VI and even IV–VI metal chalcogenide nanocrystals or endow them with dopant-dependent optoelectronic characteristics.^13−20^ To initiate a notable phase transition in extended matrices at low temperatures and reasonable yield variance, heterogeneous ion replacements at the solid–liquid interface and bulk ion diffusion need a significant acceleration.^21^ If achieved, programmable modifications of the cation sublattice may also yield TF structures with functionality that direct synthesis cannot offer.^18,20^

To date, addressing kinetic limitations in multievent reactions is a pressing issue that remains a nontrivial task faced by specialists in various fields. Regardless of the scale of resulting materials being nano or bulk, surmounting these challenges is crucial for understanding and controlling the mechanistic pathways of solid-state ion exchange in nonequilibrium conditions.^13,22^ Recent studies suggest that defect-associated protocols may contribute to the performance of a wide range of processes realized in semiconductors.^1,4^ Importantly, cationic vacancies generated on the surface of nanocrystals using certain ligands with high affinity to host cations help overcome kinetic energy barriers during the transformation of material systems I–III–VI_2_ into II–VI.^22^ Similarly, trioctylphosphine reduces the activation energy to replace Ag(I) with highly charged Bi(III), facilitating the conversion of colloidal Ag_2_S matrices into AgBiS_2_.^15^ Despite different attempts to synthesize V–VI nanomaterials doped with aliovalent Ag(I) or Cu(I) ions using cation exchange fundamentals, the transition from binary V–VI templates to ternary I–V–VI_2_ compounds has not been achieved. Beyond the nanoscale and when I–V–VI_2_ material systems are developed on substrates, overcoming thermodynamic and kinetic limitations to achieve sufficient conversion efficiency necessitates disturbances in the defect structure of initial matrices much more complex than those typically suggested for nanocrystals. In addition, powerful auxiliary tools may also be required in cation exchange solutions.^23^

Here, we report a novel synthetic strategy based on defect engineering and liquid phase design capable of intimidating rapid and highly effective cation exchange transformations at the scale of TFs. We show the viability of the proposed strategy by incorporating Ag(I) cations into about 800 nm thick TF matrices of Sb_2_X_3_ (X = S, Se). This process represents the most complicated and poorly studied multistage transition from V–VI to I–V–VI_2_ that has never been experimentally demonstrated with sufficient reaction yield in extended solids. We also show that incorporating anion vacancies and Ag_Sb_V_X_ complex defects into polycrystalline matrices of Sb_2_X_3_ triggers the replacement of Sb(III) with Ag(I) over a long distance at low temperatures. Accumulation of Ag atoms at doping levels manipulates the energy structure of Sb_2_Se_3_ films, resulting in the appearance of photoluminescence in the visible and near-infrared regions. This reveals a great potential for the proposed strategy to adjust radiative recombination pathways in TF structures. Apart from that, stretching the Lewis acidity of auxiliary chemicals in a liquid phase enables inverting upper matrix layers into AgSbX_2_ and achieving the desired accuracy of reaction yield when converting Ag precursors into a solid-phase product. Deep phase transformation causes a substantial redshift in optical band gap values and the manifestation of significant antimicrobial activity under visible light. Similar defect-engineered cation exchange approaches can be applied to functionalizing a wide range of metal chalcogenide materials.

## 2 Results and Discussion

### 2.1 Matrix Defects as Host Sites for Foreign Ions

The effect of introducing defects into orthorhombic Sb_2_Se_3_ on the intercalation of monovalent foreign ions was first probed using density functional theory (DFT). Our reference reactions confirmed that stoichiometric and highly crystalline Sb_2_X_3_ (X = S, Se) TFs require external driving forces to initiate any adequate cation replacement. We have previously demonstrated that monovalent activations by H, Cl, and OH of sulfide (selenide) matrices introduce numerous charge-compensating metal site vacancies, facilitating intercalation/deintercalation of foreign ions such as polyvalent Sn or trivalent Sb.^23,24^

To design high-yield aliovalent cation replacements to produce efficient thin-film materials, we determined the preferred positioning of silver atoms in Sb_2_Se_3_. The behavior toward intercalation of functional monovalent ions such as Ag and Cu in semiconducting chalcogenides is of interest to numerous light-harvesting applications. In general, migration of atoms in defect-free matrices is energetically less favorable than migration in defective matrices since there is more space in defective matrices, either in the form of vacancies into which extrinsic atoms can jump or in the form of additional space between atomic columns due to the presence of interstitials. Force-field molecular dynamics (FF-MD) simulations of cation exchange in chalcogenide systems show that the extrinsic cation species preferably reside at substitutional rather than interstitial positions. This implies that self-defects such as vacancies are essential to the migration mechanisms.^25,26^ To investigate the hopping energetics using FF-MD for the current Ag: Sb_2_Se_3_ system would require a separate simulation study. Here, by means of DFT, we calculate the formation energies of Ag atoms at Sb substitutional sites (substitutional point defects) and combinations thereof with adjacent Se vacancies (i.e., defect dimers), where the density of states (DOS) of the fully relaxed defect configurations may reveal midband gap defect states that can be compared with experimental photoluminescence spectra. For this, we considered a model for Sb_2_Se_3_ featuring multiple types of imperfections (Figure S1, Table S1). The defect-free Sb_2_Se_3_ crystal structure poses two inequivalent Sb positions and three inequivalent Se positions, yielding multiple possibilities to create single-point defects, dimers, or trimers (Table S1). The difference in energy between the vacancies of Sb1 and Sb2 is minimal, indicating their almost equal probability of formation. The Se1, Se2, and Se3 vacancies also have comparable formation probabilities with an energy discrepancy of less than 0.05 eV. Modeling a practical substitution of Sb(III) by Ag(I), our calculations show a preference of 0.3 eV for Ag atoms occupying the Sb2 site compared to the Sb1 site. Of more complex defects where a substitutional Ag atom is combined with a Se vacancy, Ag_Sb1_V_Se2_ was found to be the most favorable, closely followed by Ag_Sb1_V_Se1a_ and Ag_Sb2_V_Se2_ with energy differences of less than 0.1 eV (Table S1). In a nonstoichiometric Sb_2_Se_3_ system, Ag cations have no preference for occupying either Sb1 or Sb2 sites when combined with a Se vacancy. From a thermodynamic perspective, dimer defects of metal atoms paired with Se vacancies in metal selenide matrices suggest the facilitated introduction of third-party monovalent ions.

### 2.2 Ag(I) Doping and Electronic Behavior

To investigate this hypothesis derived from our DFT calculations, we fabricated TF matrices by converting SnSe into Sb_2_Se_3_. The earlier described synthetic strategy yields crystalline and stoichiometric Sb_2_Se_3_ samples.^23^ In this work, we introduced a relatively high concentration of selenium vacancies into the orthorhombic Sb_2_Se_3_ framework,^27^ as can be expected from the selenium deficiency caused by partial leaching of Se ions during thermal treatment in glycerol (Figure S2a).

The activated films were then exposed to different Ag-containing media to introduce Ag(I) dopant. Multiple data analysis was employed to evaluate the behavior of the formed defective matrices in absorbing Ag ions in the presence of auxiliary chemicals (Table S2). In the presence of NaHCO_3_, Sb_2_Se_3_ films accumulated Ag ions at the doping levels. Energy dispersive X-ray spectroscopy revealed Ag concentrations below the detection limit when using a detector integrated into a scanning electron microscope (EDX-SEM) and around 1.5 atom % when using a detector in a transmission electron microscope (EDX-TEM) (Figure S2b). Structural characteristics of the pristine Sb_2_Se_3_ and those samples treated in the Ag: NaHCO_3_ solution were investigated using X-ray diffractometry and Raman spectroscopy (Figure S2d,e). Both samples exhibit diffraction patterns consistent with a single phase of Sb_2_Se_3_ (ICDD database file 01-089-0821) that can be ascribed to the orthorhombic Sb_2_Se_3_ structure. Close inspection of X-ray diffraction (XRD) patterns (Figure S2d) enclosed a noticeable shift of the diffraction peaks to lower angles for the Ag-doped sample. Such a shift is anticipated for an ideal crystal when smaller Sb^3+^ ions (ionic radius: 76 pm) are substituted by bigger Ag^+^ ions (ionic radius: 115 pm).^23,28^ Deconvoluted Raman spectra show six unaltered peak positions, evidencing no changes introduced to the crystal structure by incorporating Ag at low concentrations (Figure S2e).

**Figure 1:**
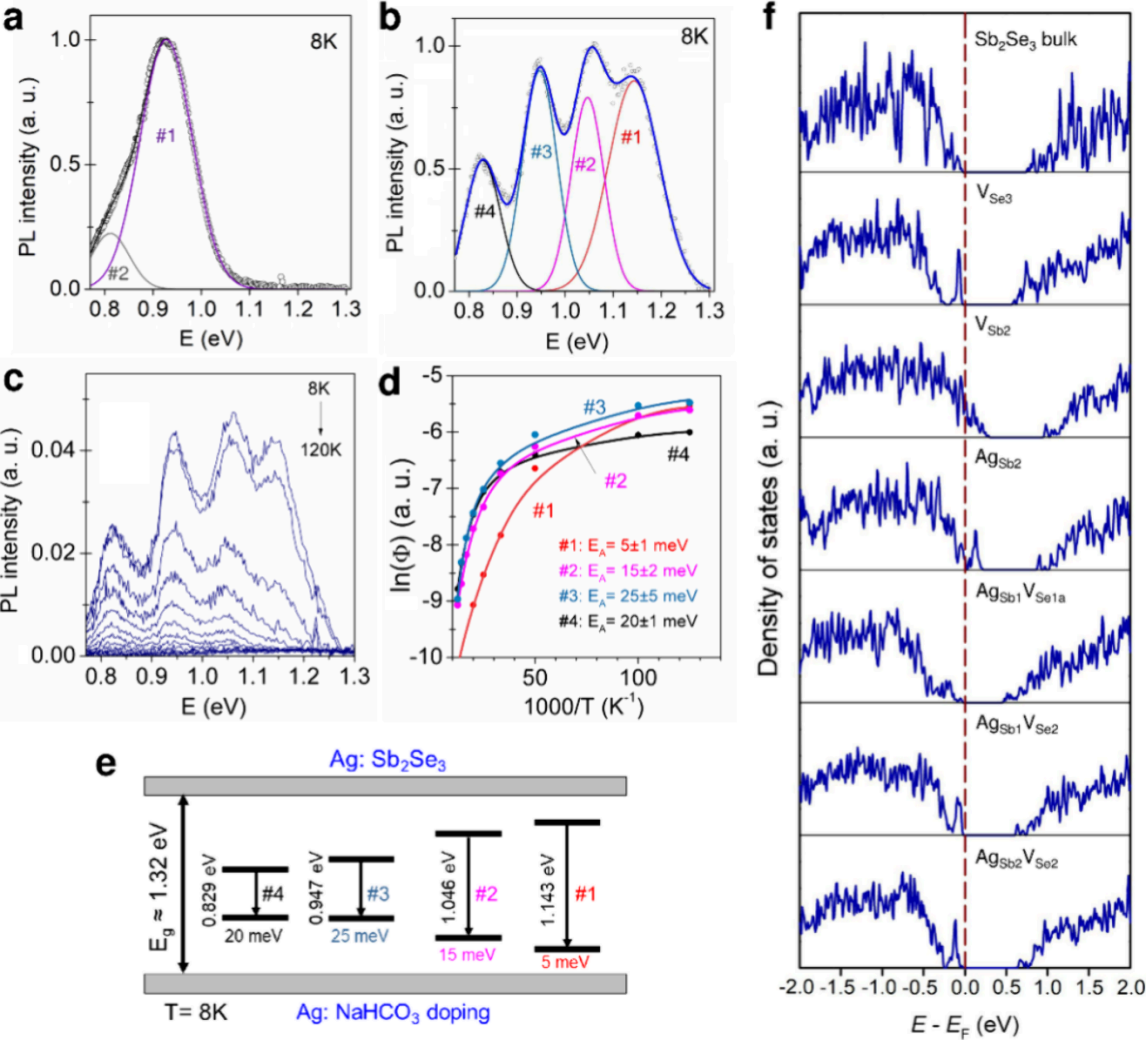
Experimental low-temperature photoluminescence (PL) spectra recorded for samples (a) pristine Sb_2_Se_3_ and (b) Ag-doped Sb_2_Se_3_ derived from the Ag: NaHCO_3_ solution. PL spectra of pristine and Ag-doped Sb_2_Se_3_ samples were fitted using the Gaussian function. (c) Temperature dependence of PL spectra for Ag-doped Sb_2_Se_3_ sample. (d) Arrhenius plots of integral intensity for PL bands and fitting results obtained using eq S1. Dots represent experimental data, and lines represent fitting data. (e) Recombination model for Ag-doped Sb_2_Se_3_ sample at 8 K. (f) Density of states for Sb_2_Se_3_ bulk and Sb_2_Se_3_ with various defects, as listed in Table S1. The energy is plotted with respect to the Fermi energy *E*_F_. In several cases, a tail of very low density of states stretches from the valence band into the band gap, which can sometimes lead to the appearance of the top of the conduction band at energies lower than the computed Fermi level (at *E* = 0.0 eV).

The effects caused by incorporating Ag at doping levels were further explored using photoluminescence (PL) spectroscopy. Figure 1a,b show experimental low-temperature PL spectra of the pristine and Ag-doped Sb_2_Se_3_ samples. The pristine Sb_2_Se_3_ film exhibits a dominant emission peak at around 0.9 eV and a less pronounced one near 0.8 eV. Silver incorporation induces drastic changes in the PL data, resulting in the appearance of four distinct bands (Figure 1b). The temperature dependence of these PL bands demonstrates very fast quenching without discernible alterations in the peak positions (Figure 1c). Such behavior with rising temperature predicts low thermal activation energies required for all PL bands. Using Arrhenius plots (Figure 1d) and eq S1, we established that all band-related activation energies were no greater than 30 meV.^29,30^ Considering the positions of the detected emission peaks, which lie quite distant from the low-temperature band gap energy of 1.32 eV reported for Sb_2_Se_3_,^31^ and the obtained modest thermal activation energies, all the PL bands observed for both pristine and Ag-doped Sb_2_Se_3_ samples most probably originate from deep donor-deep acceptor (DD-DA) pair recombination.^32,33^ A similar model was recently suggested for PL bands in polycrystalline Sb_2_Se_3_.^29^

The electron (hole) wave function at the deep donor (acceptor) level is known to be highly localized. Therefore, there is practically no overlapping of carriers’ wave functions and no observable recombination emission for more distant pairs. All single defects in Sb_2_Se_3_ are relatively deep and, consequently, can form DD-DA pairs.^34^ When paired, single acceptor and donor defect levels are pushed toward the band edges, substantially decreasing the thermal activation energies of these complexes. Numerous possibilities for forming single-point defects and their combinations in the Sb_2_Se_3_ crystal lattice (Figure S1) can provide a wide range of intersite distances. The shortest distance *r* = 0.2588 nm is between Sb2 and Se1 sites, corresponding to the Coulomb energy of 232 meV calculated employing eq S2 and ε value of 24.^35,36^ Slightly greater distances are also conceivable between different Sb and Se lattice sites.

Besides, intercalating Ag atoms into the matrix can introduce additional emission options that are not typical for pure Sb_2_Se_3_. Examinations of DOS to clarify the electronic structures of supercells under applied defects suggest metallic behavior in the presence of single-point Sb vacancies. The material remains a semiconductor with a slightly reduced band gap when introducing single Se vacancies (Table S1). Partial Ag occupation of metal sites also leads to metallic behavior (Figure 1f), practically suppressing photoluminescence. However, when a substitutional Ag atom is merged with a Se vacancy in Ag_Sub_V_Se_ complex defects, the semiconducting character of formed Sb_2_Se_3_ is preserved. The most favorable Ag_Sb2_V_Se2_ and Ag_Sb1_V_Se1a_, with their lowered band gap values (Table S1), are likely responsible for additional PL peaks 1 and 2, respectively (Figure 1f). At the same time, the construction of defects with much more complex compositions and atomic environments during the intercalation of Ag atoms may cause the amplification of PL peak 4 with its simultaneous slight shift to higher or lower energy values compared to PL peak 2 observed for pristine samples. The formation of Ag_Sb_V_Se_ complex defects can be considered energetically favorable from a charge balance point of view. If Ag atoms replace Sb atoms, an atom with a valence state of +3 is replaced by an atom with a valence state of +1, leaving the material short of two electrons that otherwise would be donated to the Se anions. However, the Ag_Sb_V_Se_ defect can be considered as a missing Sb^3+^Se^2–^ unit with a net valence state of +1, which is replaced by an Ag ion with a valence state of +1, rendering this defect charge-neutral to the rest of the material and resulting in less strain and lower migration barriers.

The experimental observation of notable substitution of Sb for Ag in TFs under Sb-rich conditions is an interesting phenomenon, given the common belief that cation vacancies are mainly responsible for contributing to the guest-cation introduction and host-and-guest-cation diffusion during kinetically unfavored cation exchange reactions.^16,22^ Such behavior can be explained using the fundamentals of the hard–soft acid–base theory. Real polycrystalline thin film materials possess a complex defect structure with a variety of 0D, 1D, 2D, and even 3D defects that may offer numerous oxidation states for Sb atoms, deviating from that in a perfect crystalline arrangement. Considering Se anion frameworks, generated Sb-rich conditions may provide Sb^3+^ in perfect crystalline arrangement, Sb^2+^ in Sb_2_Se_2_V_Se_, Sb^+^ in somewhat like Sb_2_SeV_Se_V_Se_, Sb^0,^ and V_Sb._ With decreasing the oxidation state, the softness of acids is increased, giving the Lewis acidity/hardness trend: Sb^3+^ > Sb^2+^ > Sb^+^ > SbV_Se_ > Sb^0^ ≈ V_Sb_. In such a series, the softer the defect, the easier it can be replaced with soft Lewis acids such as Ag ions. Thus, SbV_Se_ complexes are much softer than Sb^3+^, and their presence may promote energetically unfavored Sb-to-Ag cation exchange reactions.

### 2.3 Deep Matrix Transformation

**Figure 2:**
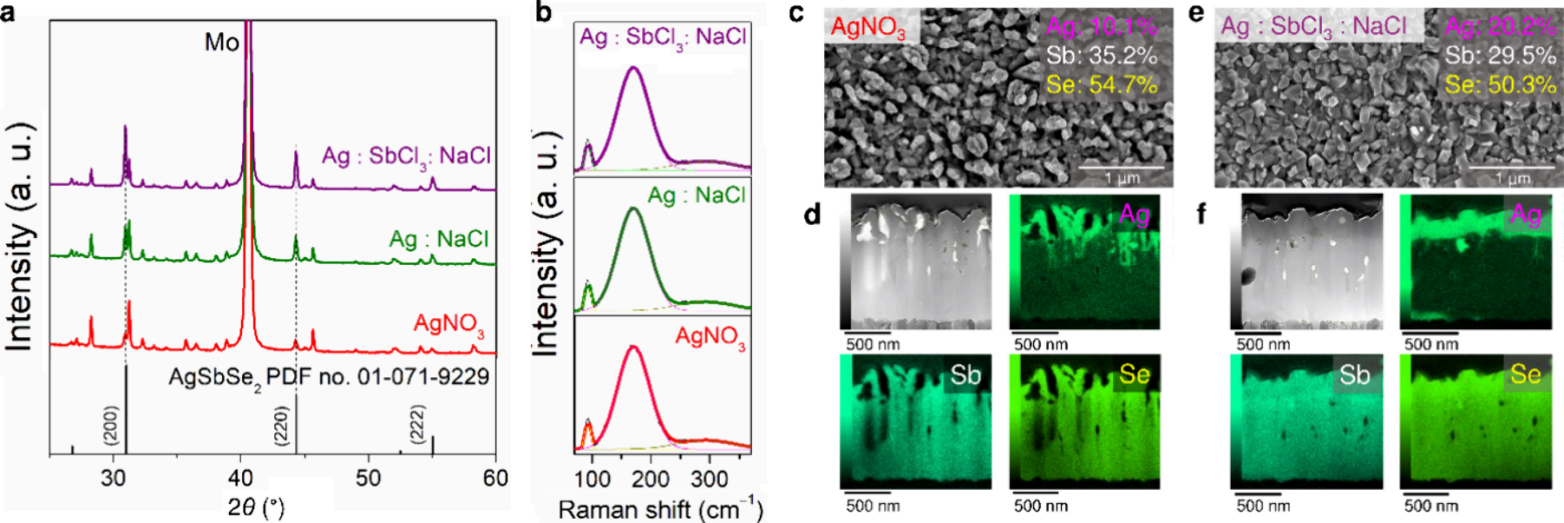
(a) XRD patterns and (b) Raman spectra recorded for inverted Ag: Sb_2_Se_3_ samples derived from AgNO_3_, Ag: NaCl, and Ag: SbCl_3_: NaCl solutions. (c, e) SEM images, TEM/EDS analysis, and (d, f) cross-sectional STEM images with corresponding EDS elemental mappings for Ag: Sb_2_Se_3_ samples derived from AgNO_3_ and Ag: SbCl_3_: NaCl solutions. Structural and compositional characteristics were averaged using measurement data from three independent repeats.

PL data manifested severe changes in the defect structure of Sb_2_Se_3_ after Ag doping, suggesting a matrix ability to subsequent cation replacement that is even more pronounced than in the pristine state. Assuming this, we tested the Ag: Sb_2_Se_3_ films formed in the previous step to append Ag(I) cations further using three distinct solutions (Table S2). After secondary Ag incorporation, the obtained samples demonstrate well-resolved X-ray diffraction peaks attributed to the cubic AgSbSe_2_ phase (ICDD database file 01-071-9229) and the remaining orthorhombic Sb_2_Se_3_ phase (Figure 2a). Stretching the Lewis acidity of auxiliary chemicals in solutions increases the peak intensities of AgSbSe_2_ and proportionally diminishes the XRD reflections of Sb_2_Se_3_. This correlation accompanies a remarkable growth in the crystallite size of the AgSbSe_2_ phase (Table S3). Such a surprising dependence may imply a higher inversion degree of Ag-doped matrices. Surface-sensitive Raman spectroscopy measurements also show a colossal difference between the crystal structures of Ag-doped (Figure S2e) and inverted samples (Figure 2b). Experimental diffractograms and Raman spectra recorded for the inverted samples at room temperature do not contradict the data simulated for the rock-salt structure of AgSbSe_2_ (Figure S4).

Broad spectroscopic assessment of the chemically silverized samples was conducted by EDX-SEM compositional analysis and verified locally in scanning transmission electron microscopy (STEM) mode (Figures 2c,e, and S5). Both techniques demonstrate comparable Ag concentrations, indicating a high uniformity of the resulting samples formed upon proposed cation exchange processing. Quantifying Ag contents reveals atomic percentages of around 10.1 ± 0.3, 14.5 ± 0.3, and 20.2 ± 0.5% collected by the samples inverted in AgNO_3_, Ag: NaCl, and Ag: SbCl_3_: NaCl solutions. Elemental mapping acquired at the L edge of the Ag element reveals the formation of two-layered structures with silver atoms preferentially localized in the upper layers. The EDX line scans in Figure S6 also confirm a two-zone distribution of Ag atoms. It is noteworthy that the thickness and continuity of the upper layer containing Ag increase when NaCl and especially SbCl_3_ are present in secondary cation exchange processes, which indicates a deeper transformation of the pristine state into AgSbSe_2_ (Figures 2d,f, and S5–S7). Similar compositional changes are recorded for thermodynamically unfavored conversion of Sb(III) sulfide frameworks (eq S4 and Figure S8). SEM visualization of the obtained morphologies shows compliance with a polycrystalline structure and uniform grain size distribution in the range of 100–150 nm (Figures 2c,e and S5).

**Figure 3:**
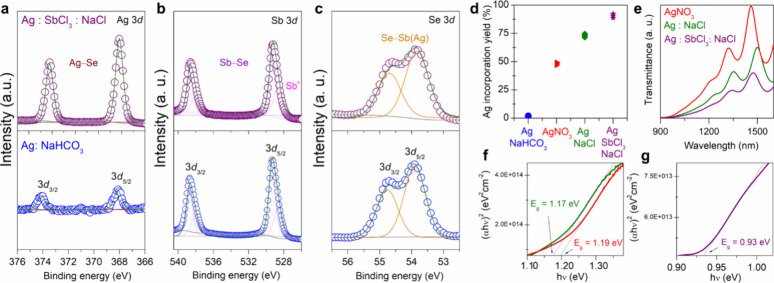
(a–c) Comparison of high-resolution spectra of Ag 3*d*, Sb 3*d*, and Se 3*d* core level peak regions of Ag: Sb_2_Se_3_ samples derived from Ag: NaHCO_3_ and Ag: SbCl_3_: NaCl solutions. (d) Accumulation yields of Ag atoms manifested in Ag: Sb_2_Se_3_ films that experienced exposure to various solutions (Table S2). Yield values were determined by the ICP-measured Ag(film): Ag (solution) mass ratio expressed in percentages. (e) Transmittance spectra and (f, g) Tauc plots for Ag: Sb_2_Se_3_ films derived from AgNO_3_ (red curve), Ag: NaCl (green curve), and Ag: SbCl_3_: NaCl (purple curve) solutions. Dotted lines represent an extrapolation of the rectilinear portion of absorption edges. Presented values were averaged using measurement data from three independent repeats.

The incorporation of Ag into Sb_2_Se_3_ was further explored using X-ray photoelectron spectroscopy (XPS). Before collecting XPS spectra, silverized samples were etched with argon cluster ions for 60 s to decrease atmospheric contaminations. The presence of Ag 3*d* peak splitting into two 3*d*_3/2_ (374.2 eV) and 3*d*_5/2_ (367.9 eV) components confirms the Ag doping and subsequent intensive accumulation of Ag in Sb_2_Se_3_ (Figure 3a). The binding energy of Ag 3*d* doublet matches that of Ag varieties with a preserved oxidation state (I).^37^ The Sb 3*d* peak (Figure 3b) is fitted with the 3*d*_5/2_ (529.3 eV) and 3*d*_3/2_ (538.6 eV) doublet using a fixed area ratio of 3:2 and a spin–orbit splitting of 9.3 eV. The peak positions in the Sb 3*d* regions correspond to the binding energy of lattice Sb^3+^ in selenide compounds.^23^ The juxtaposition of XPS spectra shows a significant increase in the peak area of Ag and a notable decrease in the peak area corresponding to Sb after adding auxiliary salts to the cation exchange solution system (Table S4). Such changes refer to the apparent replacement of Sb(III) with Ag(I), as suggested by our X-ray diffraction results and compositional alterations observed in EDX line scans. The Se 3*d* XPS spectra in Figure 3c show two typical 3d_5/2_ and 3d_3/2_ peak constituents with binding energies at approximately 53.8 and 54.8 eV, attributing to Se–Sb(Ag) bonding. Qualitative XPS analyses show that Ag doping can activate the pristine matrices for ensuing cation exchange reactions in extended solids.

Inductively coupled plasma mass spectrometry (ICP-MS) was employed to quantify Ag accumulation in Sb_2_Se_3_ matrices relative to theoretically possible concentrations. ICP-MS data show that Ag inclusion yield can be gradually increased from about 1.5% for doping processes implemented in Ag: NaHCO_3_ solutions to values exceeding 90% when multi-ion compositions displayed by Ag: SbCl_3_: NaCl solution case are used for cation replacement (Figure 3d, Table S5). The ability to control the efficient utilization of reactants on rigid substrates in such a simple and cheap way represents a big step forward in optimizing industrial processes involving expensive and/or toxic precursors.

Phase transformations occurring due to the inclusion of Ag atoms in Sb_2_Se_3_ or obvious substitution of Sb by Ag encourage a change in energy gaps. Ultraviolet–visible (UV–vis) spectroscopy was used to estimate the band edge positions of materials derived from cation exchange. Tauc plots show a coinciding optical band gap of 1.28 eV for the pristine Sb_2_Se_3_ and Ag-doped sample derived from the Ag: NaHCO_3_ solution (Figure S2f). After preliminary doping, the secondary incorporation of Ag causes a prominent redshift of the absorption edge (Figure 3e–g), with band gaps of 1.19, 1.17, and 0.93 eV for films obtained from AgNO_3_, Ag: NaCl, and Ag: SbCl3: NaCl solutions, respectively. Such a reduction in band gap values is directly associated with a gradually increased silver content and enlarged inversion degrees of the stating Ag-doped Sb_2_Se_3_ point. This is because the valence band consisting of Se *4p* orbitals in Sb_2_Se_3_ is partially or entirely hybridized with Ag *5s* with a higher electronic potential, which leads to a band gap narrowing (Figure 1f, Table S1). These results indicate that advances in introducing defects and supplements in solutions play a critical role in controlling metastable products of cation exchange, especially those formed at moderate temperatures below 250 °C. The one-step doping strategy using an Ag-containing precursor facilitates a follow-up inversion of extended solids with controllable compositions. This discovery prompted us to carefully study the features implemented in our solution systems.

### 2.4 Solution Behavior

The complexation of metal salts to glycerol raises the question about the nature and strength of this binding, which determines the availability of cations for solid matrices during cation exchange processes. Multisalt compositions make such determinations even more complicated. To reveal at least the groups bound to cations, we compared pH units obtained using pH indicator strips and Raman spectroscopic data collected from solutions specified in Table S2. Pure glycerol exhibits a pH of around 6–7 (Figure S10). Considering the thermodynamic p*K*_a_ value of 14 for water and a very similar value of 14.4 for glycerol^38^ and the graduation and accuracy of pH indicator strips as 1 pH unit, pure glycerol is about neutral. Dissolving AgNO_3_ at 0.224 mM results in a slightly acidified solution with pH values between 4 and 5. Similar pH values are obtained for a 2.17 mM NaCl glycerol solution. Solutions of glycerol containing SbCl_3_ at 44 mM are strongly acidic with a pH of around 2. A simple evaluation of recorded pH parameters suggests that metal salts are likely to interact with glycerol molecules with realizing protons.

**Figure 4:**
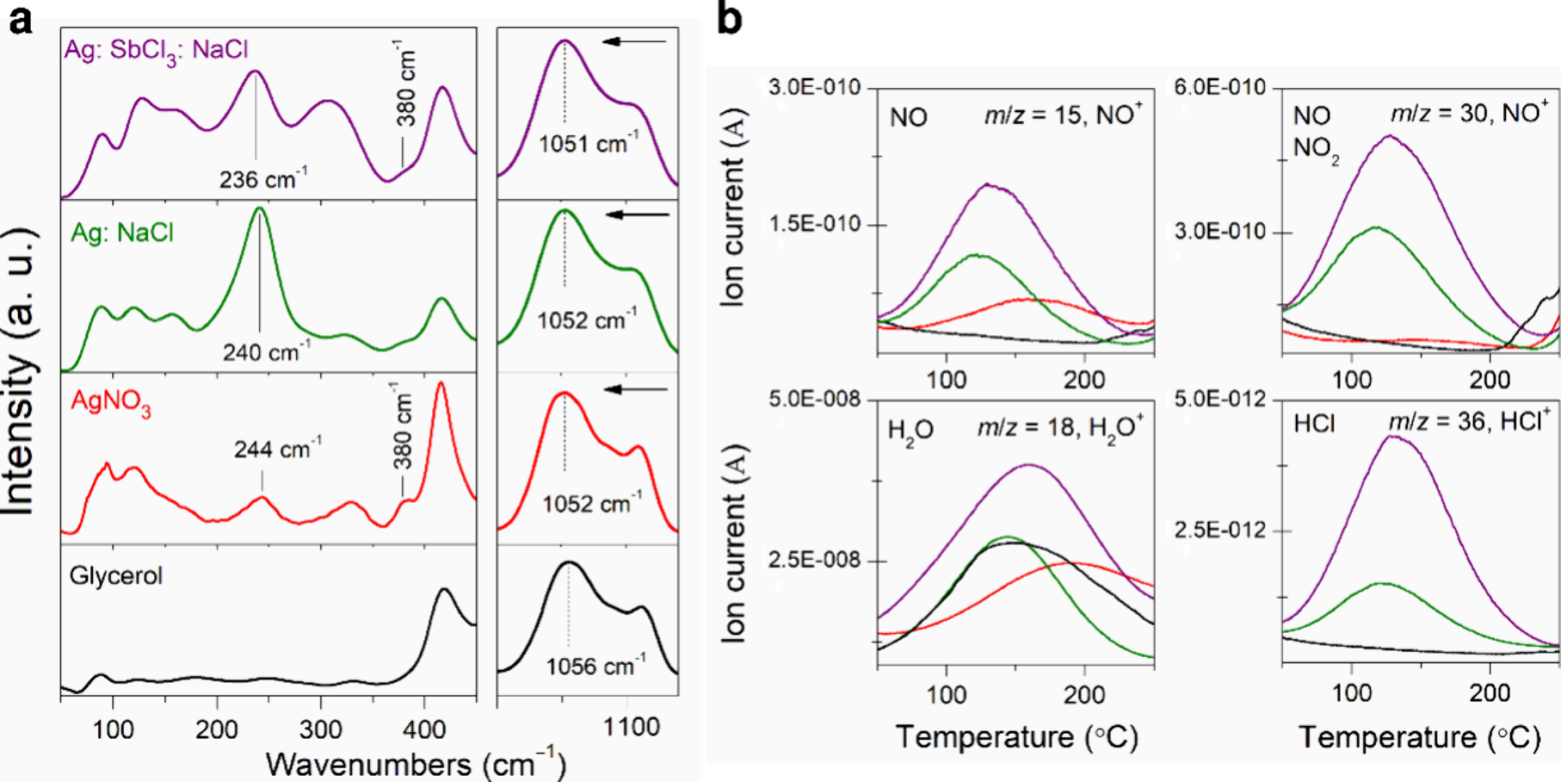
(a) Raman spectra for pure glycerol and Ag-containing solutions in the presence of supportive chemicals. (b) Gas evolution profiles of anion-derived gaseous species represented by their characteristic mass spectroscopic ion fragments from pure glycerol (black curve) and solutions of AgNO_3_ (red curve), Ag: NaCl (green curve), and Ag: SbCl_3_: NaCl (purple curve). Gaseous species are measured by the TG/EGA-MS system in flowing argon with an Ar flow rate of 60 mL min^–1^ and heating rate of 10 °C min^–1^. The initial masses of solutions were 26.7 mg (glycerol), 26.7 mg (AgNO_3_), 33.7 mg (Ag: NaCl), and 37.8 mg (Ag: SbCl_3_: NaCl). Presented values were averaged using measurement data from three independent repeats.

Analyzing the Raman spectrum of plain glycerol and those collected from solutions of a single salt source and their mixtures shows the coordination of metal cations to glycerol molecules (Figures 4a,b and S11). The formation of metal-containing complexes typically results in changes or shifts in the vibrational frequencies of the ligand compared to its free state.^39,40^ When AgNO_3_, NaCl, and SbCl_3_ and their mixtures are dissolved, νCO vibrations located at 1112 cm^–1^ (C2) and 1056 cm^–1^ (C1, C3) in pure glycerol are shifted to slightly lower frequencies to 1108 and around 1052 cm^–1^, respectively, indicating the distinct coordination modes and the presence of deprotonated glycerol species.^41^ Raman spectra of the solutions containing metal sources show significant similarity in the low-frequency region of 50–400 cm^–1^. Several spectral features cannot be attributed to undissolved single salt or glycerol. (Figure 4a). Appearing the shoulder near 380 cm^–1^ for all solutions except pure glycerol indicates the interaction between metal salts and solvent. This band is positioned at too low frequencies that are too low to be assigned to partially dissociated cationic units (e.g., SbCl_2_^+^).^42,43^ Such characteristics most probably relate to anion-deficient metal species of polymeric nature resulting from acid–base interactions of metal salts and glycerol.^43^ The broad peak at about 240 cm^–1^ suggests polymeric signatures exist in our systems.^42,44^ The relatively weak doublet with altered positions at around 160 and 120 cm^–1^ is recorded for all salt-containing solutions (Figure 4a). If polymeric complex species are formed in our concentration regions, relatively featureless spectra with fairly broad bands can be expected. Such interactions further distance anionic species from the formed metal cationic units, most likely coordinating with oxygen atoms of glycerol. This may facilitate the decomposition reactions that include precursor-derived anions or gas evolution upon heating.

Assuming the conjecture above, we studied the gaseous phases using thermogravimetry evolved gas analysis (TG-EGA). We mainly concentrated on low-temperature processes since the designed multi-ion solutions are active enough to trigger substantial Sb-to-Ag replacement at temperatures below 250 °C. The TG-EGA data of pure glycerol and working solutions show dehydration and decomposition of anionic residues of precursors in the temperature region of interest (Figures 4b and S12). During mass loss events at low temperatures, TG-mass spectroscopy (TG-MS) confirms the release of H_2_O, NO, and NO_2_ as volatile decomposition products of solutions containing nitrate ions. When chlorine anions are present in the working solutions in addition to the source of silver, HCl evolution is also observed (Figure 4b). It is important to note that similar fragmentation from pure AgNO_3_ has been observed in the 300–550 °C temperature region.^45^ Thus, detecting nitrogen-containing fragments at much lower temperatures than observed for solid silver nitrate confirms its ionization in the working concentration region.

**Figure 5:**
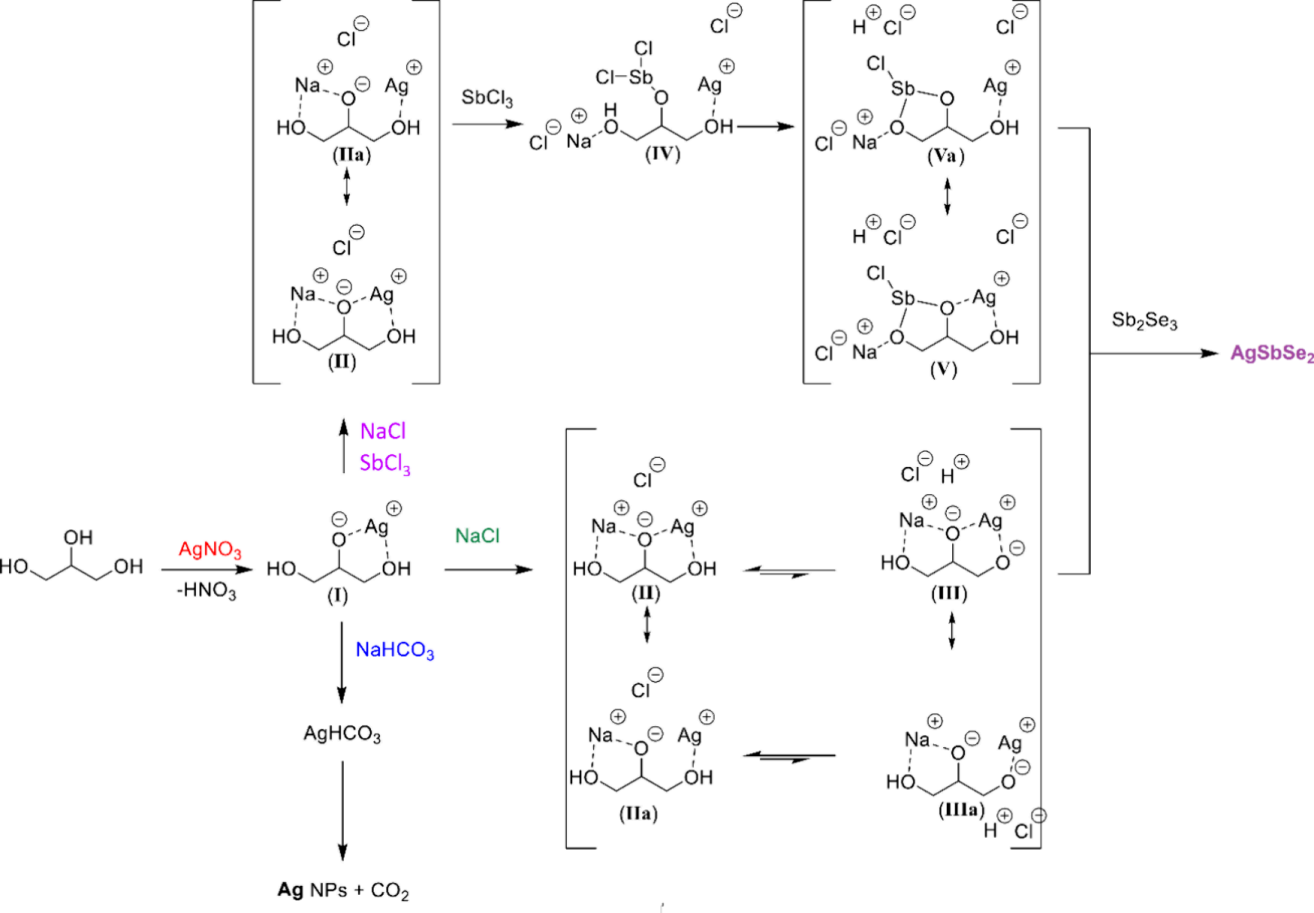
Routes for forming Ag nanostructures and controlled transformation of TF materials.

The formation of such distinct Ag: Sb_2_Se_3_ systems with dissimilar yields of Ag inclusion without adding complexing agents to glycerol solutions can be rationalized by differences in the activities of Ag cations formed in the presence of supporting ions. In general, the solvation energy of ions extracted from TFs should exceed their binding energy with the crystal lattice to ensure cation replacement and vice versa for incorporating ions. Using Ag: NaHCO_3_ solutions, we obtained Sb_2_Se_3_ samples that accumulated Ag close to doping levels. In this case, simple baking soda acts as a soft base that preferably interacts with solvated Ag cations (Figure 5, **I**) to release an intermediate AgHCO_3_. When heated, unstable AgHCO_3_ decomposes rapidly to form Ag nanoparticles (Figure S13). Thus, introducing bicarbonate anions possessing weak miscibility with Ag cations can selectively reduce their solubility, affecting their availability for cation exchange reactions and generating the conditions for doping.

The high crystallinity of pristine Sb_2_X_3_ TFs^23^ and existing energy barriers^46^ complicate direct Sb-to-Ag exchange reactions. Therefore, significant changes in the defect structure are necessary to balance driving forces. As shown above (Figure 1), pretreatment using Ag: NaHCO_3_ solutions was sufficient to prepare the samples for intensive cation substitution. Reacting Ag-doped Sb_2_Se_3_ with AgNO_3_ and, in particular, Ag: NaCl or Ag: SbCl_3_: NaCl solutions leads to the transformation of the upper layers of Sb_2_Se_3_ into AgSbSe_2_. The observation of a stepwise increase in the yield of Ag inclusions (Figure 3d) when adding NaCl or a mixture of SbCl_3_ and NaCl to solutions containing AgNO_3_ is unexpected, considering the poor solubility of AgCl that might form. However, we have not observed its precipitation in any solution containing Cl ions (Table S2). This fact and HCl release detected at temperatures even below 100 °C (Figure 4b) suggest a rather bizarre complexation. Our computational studies reveal a similar thermodynamic stability for metal–organic complexes formed upon interacting glycerol molecules with Ag and Na ions. Polymeric signatures comprising Sb atoms or partially dissociated Sb–Cl units turn out to be an order of magnitude more stable than formations comprising Ag and Na (Table S6). Thus, considering two main factors, such as concentrations of AgNO_3_ and auxiliary chemicals (Table S2) and significant differences in thermodynamic stabilities, we can conclude that Na and Sb cations compete with Ag cations, displacing them from formed complexes.

In more detail, when NaCl is added to (**I**) (Figure 5), the Na ion binds to the alkoxide and the lone pair of electrons on the free OH group (**II**). This decreases the ionic character of the bond between the alkoxide and Ag ions, weakening their interaction (**IIa**). The alcohol group bound to the Ag ion of both bimetallic complexes (**II** and **IIa)** is in equilibrium with the deprotonated form (**III** and **IIIa**). However, this equilibrium strongly favors the protonated forms (**II** and **IIa**). This accounts for the low concentration of HCl found and is consistent with the recorded pH values for the NaCl route. Complexes **II** and **III** are expected to be the most stable due to bidentate coordination with the Ag ion. Their relatively high stability determines a low reactivity of Ag ions with respect to Cl ions and supports the absence of AgCl sedimentation under these conditions.

In the route where SbCl_3_ is injected into glycerol solutions of (**I**) and NaCl, two possibilities can be realized: (i) NaCl binds first, and then a reaction with SbCl_3_ occurs, or (ii) SbCl_3_ reacts first, and NaCl interacts in the following steps. The first option is more likely since Na alkoxide (**IIa**) is expected to be the most reactive metal alkoxide available to interact with SbCl_3_. The complexation of NaCl to the Ag glycerol adduct (**I**) is analogous to the Ag: NaCl route. A reaction of intermediates (**II** and **IIa**) with SbCl_3_ is expected to occur fast, so the equilibria to (**III** and **IIIa**) will have a minor role in the Ag: SbCl_3_: NaCl system. Formation of the first Sb–O bond (**IV**) results in a release of the Cl ion, which does not explain the recorded low pH values (Figure S10). Meanwhile, the second Sb–O bond (**Va**) formation releases HCl. Due to the covalent character of Sb–O bonds, a lone pair of electrons on the O atom is considered “soft” compared to “hard” alkoxide derivatives. These “soft” lone pair of electrons now prefer the “soft” Ag ion over the “hard” Na cation. Combined with the stability of bidentate coordination, the resulting Sb Ag complex (**V**) is expected to form. Following the argumentation given for the Ag: NaCl route and no precipitation observed even under Ag: SbCl_3_: NaCl conditions, the formation of stable bimetallic complexes can explain the reduced reactivity of Ag cations for reactions with Cl ions.

In general, cation competition makes Ag(I) more accessible to solid matrices, determining a higher inversion degree of Sb_2_Se_3_ in AgSbSe_2_ when NaCl and strong Lewis acids like SbCl_3_ are injected into glycerol-based cation exchange solutions. Using Sb-to-Ag replacement as a model for detailed characterization, we schematically presented simple guidelines for synthesizing numerous chalcogenide films with a controlled yield (Figure 5). Similar customization can be widened to most noble metals, including Cu and sulfide/selenide films.

### 2.5 Antibacterial Activity of Thin Films Most Inverted into AgSbSe_2_

Each new technology and synthesized material raise the question of their applicability. Presently, the properties of AgSbSe_2_ are poorly understood, complicating the correct determination of its compliance and suitability. Although some reports demonstrated that AgSbSe_2_ powder might exhibit thermoelectric functionality,^47,48^ the properties of thin-film materials broadly are unknown. Obtaining continuous structural characteristics and the polycrystalline nature of AgSbSe_2_ TFs (Figure 2), compositional features (Figure 3a–c), and band gap energy below 1.0 eV (Figure 3g) led us to assume that the structures derived from the most high-yielding Sb-to-Ag replacement (Figure 3d) may exhibit antimicrobial activity. The most studied photoactive antibacterial agents, such as TiO_2_ and ZnO, have relatively wide band gaps of ≥3.0 eV, which limits their potential application only in areas with accessible ultraviolet (UV) light sources. Meanwhile, typical indoor spaces are illuminated by white light with a minor portion of UV radiation,^49^ requiring materials with a high absorption coefficient and a band gap lower than common oxide photocatalysts. To follow the idea of creating antimicrobial materials responsive to visible light, many different candidates with theoretically more or less appropriate optoelectronic characteristics have been tested. Anatase TiO_2_ powder polymerized with polyaniline, graphene/silver nanocomposites, and nanostructures of Cu_2_SnS_3_ or TiInCrO_6_ have displayed certain antibacterial activities toward various Gram-negative and Gram-positive bacteria when illuminated with visible light.^50−54^ Since these groups of bacteria have different cell wall structures, the observed antibacterial activity of the listed materials may be due to combinatorial effects affecting various biological structures. Considering theoretical expectations,^50−55^ experimental data above, and recorded photosensitivity and p-type conductivity (Figure S14), we assume that TFs most inverted into AgSbSe_2_ possess multimodal antimicrobial activity to be developed under visible light illumination.

**Figure 6:**
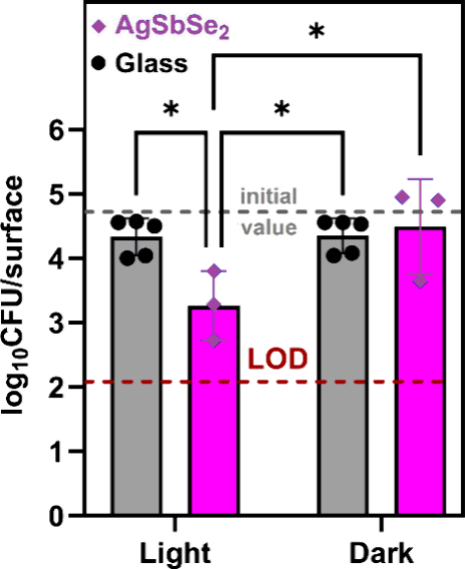
Antibacterial activity of AgSbSe_2_ layers derived from Ag: SbCl_3_: NaCl solutions toward *Escherichia coli* after 1.5 h of exposure under visible light and in the dark. The viable bacterial number is represented by log10-transformed colony-forming units (CFU) on AgSbSe_2_ TF surfaces and negative control surfaces (borosilicate glass). The initial value represents a viable bacterial number introduced to the surface at the beginning of tests. The red dotted line indicates the detection limit of the assay. Presented values were averaged using measurement data from at least three independent repeats. Statistically significant differences (*p* < 0.05) are marked with an asterisk.

Our preliminary screening with the Gram-negative bacterium *Escherichia coli* showed that 2 h exposure to AgSbSe_2_ layers under visible light inactivated more than 99% of bacteria, and the bacterial counts fell below the detection limit (Figure S15). Thus, we shortened the exposure time to quantify the antibacterial activity more accurately. After exposure for 1.5 h, *E. coli* counts decreased by 95% on illuminated AgSbSe_2_ TFs (Figure 6). The material reveals no significant antibacterial activity in dark conditions. This fact, accompanied by the lack of bacterial inhibition zones on agar plates surrounding the AgSbSe_2_ samples and negative control-like bacterial growth underneath them (Figure S16), suggests that the antibacterial activity of formed AgSbSe_2_ structures is not caused by the leaching of inhibitory species but rather by a photo assisted catalytic mechanism. Briefly, light irradiation ejects electrons from the filled valence band to the unoccupied conduction band of AgSbSe_2_, leaving holes in the electronic environment of mostly Se (Se^·–^) and promoting electrons to the environment of mostly Sb (Sb^(3–*n*)+^).^47^ Photogenerated holes at the valence band can interact with aqueous solutions, producing different reactive oxygen species (ROS: OH^·^, O_2_^·–^, HO_2_^·–^)^56^ or directly with electron-enriched sites of bacterial membranes. Photoexcited Sb^(3–*n*)+^ at the conduction band can be relaxed similarly by (i) interacting with water and generating ROS or (ii) transferring electrons to the valence band or electron-deficient centers of bacterial membranes with restoring Sb^III^ states. Both aspects could contribute to the development of antibacterial properties of AgSbSe_2_. In general, narrowband materials often require nanoscale configurations, unique architectural designs, and external stimulators to switch on synergistic effects for manifesting notable antibacterial properties. For instance, nanosheets of Sb_2_Se_3_, with their high surface-to-volume ratio and natural ability to damage bacterial membranes with sharp edges, still necessitate laser irradiation to exhibit antibacterial activity.^57^ Meanwhile, separately standing Bi_2_S_3_ nanocrystals display photoexcited sterilization only after tight contact with Ti_3_C_2_T_*x*_ MXene, which may accelerate the transfer of photogenerated charges at the interface.^58^ Visible light-induced antibacterial activity recorded for inorganic structures obtained within the routes reported here is fascinating, considering the fundamental difference between bulk materials and those nanodispersed ones that are commonly used to create disinfecting surfaces or thin films responsive under ultraviolet irradiation (Table S7). Thus, this study offers fresh insights into the high-yielding synthesis and alternative designs of optoelectronic thin film materials and demonstrates the vast potential of AgSbSe_2_ for antibacterial applications.

## 3 Conclusions

By modeling energy-hindered inversion on the scale of TFs based on replacing small highly charged cations with much larger monopositive ions, we elaborated an efficient solution-assisted strategy for converting binary metal chalcogenides V–VI into ternary I–V–VI_2_ ones, using poorly understood cation exchange process to replace Sb(III) with Ag(I) as a control within Sb_2_X_3_ (X = S, Se) TF matrices. Reference experiments, theoretical simulations, and data analyses of solid and liquid phases of cation exchange systems confirmed that introducing anion vacancies and Ag_Sub_V_Se(S)_ complex defects into Sb_2_X_3_ TFs triggers energy-unfavored cation exchange reactions. Concurrently, supportive agents within various Lewis acidity can affect the reaction yield on substrates, approaching 90% of the theoretical capacity to insert Ag atoms into defect-activated Sb_2_X_3_ frameworks and inverting top layers into AgSbX_2_ when cation exchange solution systems are rationalized to Ag: SbCl_3_: NaCl composition. The presented strategy opens up a new synthetic dimension that is unattainable for common routes to initiate defect-mediated functionalization and transformation of bulk materials with acceptable yield accuracy. Moreover, obtained structures with aliovalent cation substitution possess a potential for optoelectronic applications; in particular, TF samples most heavily inverted into AgSbSe_2_ using Ag: SbCl_3_: NaCl cation exchange solution systems exhibited significant antibacterial activity after a 1.5 h span in visible light.

## 4 Experimental Methods

### 4.1 Synthesis and Chemical Silverization

Original Sn_2_X_3_ (X = S, Se) TFs were inverted from sputtered stoichiometric tin(II) sulfide/selenide (SnS/SnSe) layers using a process described in our previous report.^23^ Briefly, SnS/SnSe TFs were immersed in 44 mM solutions of SbCl_3_ for 17 min. After inversion, samples were additionally heated in pure glycerol for 30 min, then washed with deionized water and dried using compressed air. Chemical silverization was also performed using silver nitrate (AgNO_3_) as a silver source. Silver-containing solutions were prepared by dissolving a proper amount of AgNO_3_ in glycerol to reach 0.224 mM. All supportive chemicals were added to the solution systems after the complete dissolution of AgNO_3_ to avoid precipitations of AgCl. The resulting concentrations of all chemicals obtained in solutions are specified in Table S2. Silver incorporation reactions using the solutions described above were carried out for 1.5 h. All inversions and heat treatments were performed in open systems at around 210 °C. The obtained samples were washed, dried, and stored in ambient conditions.

### 4.2 Structural Characterization

The crystal characteristics were studied by X-ray diffractometry (Rigaku Ultima IV) using Cu Kα1 radiation operated at 40 kV and 40 mA. Changes in crystal structures were also examined using Raman spectroscopy (Horiba’s LabRam HR800) and a green laser. Morphologies and elemental compositions were investigated by scanning electron microscope (Zeiss Merlin) with a Bruker EDX-XFlash6/30 detector at 3 and 20 kV acceleration voltages. The PL spectra were measured using a 0.64 m focal length single grating (600 mm^–1^) monochromator under the excitation of a 442 nm line from a He–Cd laser. The temperature-dependent PL spectra were collected by cooling the sample in a closed-cycle He cryostat to ∼8 K and then heating it progressively to 300 K. Chemical states of the elements were determined by X-ray photoelectron spectroscopy (XPS, Kratos Axis Ultra DLD) using Al Kα X-ray source. Spectra were calibrated assuming the C 1s peak at 284.6 eV. XPS profiles were acquired by etching the samples with 4 keV (0.1 mA cm^–2^, 60 s) argon cluster ions. Optical bandgap values were determined using Tauc plots from the transmittance data collected by UV–vis spectrophotometer (Shimadzu UV-1800) at room temperature. The STEM images and EDS mapping were recorded using a JEOL JEM-2200FS microscope operated at 200 kV and 8–15 μA. Cross-sectional samples were prepared with an 80–100 nm thickness using focused ion beam (FEI Helios 600 DualBeam SEM/FIB) etching.

### 4.3 Density Functional Theory Calculations

Density functional theory (DFT) calculations were conducted to estimate the structural relaxation and electronic properties of Ag-doped Sb_2_Se_3_ employing the plane-wave Vienna Ab-initio Simulation Package code.^59,60^ Interactions between core and valence electrons were described using the projector augmented wave (PAW) method with cores of [Kr] for Sb, [Kr] for Ag, [Ar] for Se, and [Ne] for Na.^61,62^ The computations employed the generalized gradient approximation of Perdew, Burke, and Ernzerhof (PBE).^63^ The D3 Grimme dispersion correction with zero damping was applied to account for van der Waals interactions in Sb_2_Se_3_.^64^ To simulate cation replacement, we first performed a full geometry optimization on a perfect 3 × 1 × 1 Sb_2_Se_3_ supercell (60 atoms) using a 12 × 4 × 4 k-point mesh and an energy cutoff of 400 eV for wave functions and 560 eV for augmentation functions. The structure was optimized to achieve energy convergence at each atom within 0.5 meV. This approach provided accurate lattice parameters of *a* = 4.019 Å, *b* = 11.459 Å, and *c* = 12.047 Å, which are consistent with the experimental values of 3.988, 11.662, and 11.805 Å.^65^ Defect intercalations were calculated using a 3 × 1 × 1 supercell to obtain an approximate cubic shape for minimizing defect–defect interactions of adjacent cells due to the applied periodic boundary conditions. Each supercell contained approximately 60 atoms, depending on the defect configuration (Table S1).

### 4.4 Solution Characterization

Major species in the formed solutions containing supportive chemicals were determined using Raman spectroscopy. Solution thermal stability and gas release were simultaneously recorded using thermogravimetric analysis (SetSys-Evo 1600) and mass spectrometry (OmniStar) at a heating rate of 10 °C min^–1^ from room temperature to 500 °C under argon flow. The ion currents of the selected mass/charge (*m*/*z*) numbers were monitored in the mode of multiple ion detection (Quadera version 4.20) with a data acquisition time of 1 s for each channel. The most favorable structures of the complex compounds presumably formed in glycerol solutions were optimized using the ab initio quantum chemistry program package (ORCA).^66^ The calculations adopted the Hartree–Fock approach using the def2-SVP set for wave functions. The silver content accumulated in the sample during cation exchange concerning the initial silver concentration in glycerol solutions was estimated using inductively coupled plasma mass spectrometry (iCAP Qs ICP-MS).

### 4.5 Antibacterial Tests

Antibacterial testing was carried out for AgSbSe_2_ samples obtained using the most high-yielding procedure denoted as Ag: SbCl_3_: NaCl. For testing, inverted and thermally treated Sb_2_Se_3_ samples were cut into 25 mm × 25 mm pieces, silverized as shown in Figure S15, washed with water and 70% ethanol, then dried.

Antibacterial activity was tested against *Escherichia coli* DSM 1576 (syn ATCC 8739) using a modified ISO 22196:2011 standard.^67^*E. coli* cells were propagated on LB (yeast extract 5 g·L^–1^, tryptone 10 g·L^–1^, NaCl 10 g·L^–1^) agar plates overnight at 37 °C. Bacterial inoculum was prepared by scrapping cells from agar plates with a sterile inoculation loop and suspending them in 500-fold diluted nutrient broth (NB) with a final concentration of meat extract 0.006 g·L^–1^, peptone 0.02 g·L^–1^, and NaCl 0.01 g·L^–1^. The cell density of bacterial inoculum was photometrically adjusted to 2.4 × 10^6^ colony forming units (CFU) per ml. 25 μL of the bacterial inoculum was pipetted onto the surface of AgSbSe_2_ samples, followed by covering with 20 mm × 20 mm polyethylene film (Etra OY, Finland). Borosilicate glass (Corning Inc., USA) of 25 mm × 25 mm was used as an inert negative control. The viable count on each surface ranged between 1.5 × 10^4^ and 6.0 × 10^4^ CFU cm^–2^. After inoculation, all samples were incubated at room temperature and relative humidity above 90% in a climate chamber (Climacell EVO) for 1.5 h either in dark conditions or exposed to visible light (Philips TL-D 15*W*/840) with a photon flux density of 150 μmol m^–2^ s^–1^ at 400–700 nm as measured by PAR Quantum Radiometric Probe (Delta Ohm). To stop exposure, the samples were submerged into 20 mL of the SCDLP toxicity-neutralizing medium (casein peptone 17.0 g·L^–1^, soy peptone 3.0 g·L^–1^, NaCl 5.0 g·L^–1^, K_2_HPO_4_ 2.5 g·L^–1^, glucose 2.5 g·L^–1^, lecithin 1.0 g·L^–1^, Tween80 7.0 g·L^–1^) and vortexed for 30 s to detach the bacteria. After a serial dilution in phosphate-buffered saline (PBS), 20 μL of the *E. coli* dilutions were drop-plated onto LB agar plates. In addition, 500 μL of undiluted wash-off in SCDLP was plated to separate LB agar plates. Plates were incubated at 37 °C for 16–18 h, and the viable counts as a measure of a countable number of bacterial colony-forming units (CFU) were determined within each drop or plate. Results were expressed as log_10_-transformed CFU counts on a surface, and the value of 2.07 was determined as a limit of detection (LOD) of the method (≥3 colonies per surface). At least three biological repeats were performed for each surface and condition. Statistical analysis of log-transformed viable counts was performed using GraphPad Prism 9.5.0 Software. Two-way ANOVA followed by Tukey’s multiple comparisons test at α = 0.05 was used.

## Data Availability

The paper and/or the Supporting Information contain all the data needed to evaluate the conclusions. The authors may provide additional data related to this paper upon request.

## Supporting Information Available

Schematic illustration and DFT outcomes of defect states for bulk and partially substituted Sb_2_Se_3_; details on cation exchange solution systems; phase composition, optical and excitation power dependence of PL data for doped samples; simulated data for AgSbSe_2_; compositional and structural data for inverted samples; solution characterization data; modeled complex compounds formed in cation exchange systems; fabrication setup and photoelectrochemical characteristics of inverted structures on substrates; data on preliminary screening with the Gram-negative bacterium *Escherichia coli*; and comparison of most common agents to deactivate bacteria under illumination (PDF)
