# Supplementary material for: Efficient Defect-Driven Cation Exchange beyond the Nanoscale Semiconductors toward Antibacterial Functionalization
Source: ACS Appl Mater Interfaces. 2024 Oct 30;16(45):62871–82. doi: 10.1021/acsami.4c11425 (PMC11565473; doi:10.1021/acsami.4c11425)
Supplement: Supplementary file 1 — am4c11425_si_001.pdf [file am4c11425_si_001.pdf]

Supporting Information

Efficient defect-driven cation exchange beyond the nanoscale semiconductors toward antibacterial functionalization

Svetlana Polivtseva[1*], Olga Volobujeva[1], Ivan Kuznietsov[1], Reelika Kaupmees[1], Mati Danilson[1], Jüri Krustok[1,2], Palanivel Molaiyan[3], Tao Hu[3], Ulla Lassi[3], Mihhail Klopov[2], Heleen van Gog[4], Marijn A. van Huis[5], Harleen Kaur[6], Angela Ivask[6], Merilin Rosenberg[6], Nicholas Gathergood[7], Chaoying Ni[8], Maarja Grossberg-Kuusk[1].

[1]School of Engineering, Department of Materials and Environmental Technology, TalTech, Ehitajate tee 5, 19086 Tallinn, Estonia

[2]School of Science, Department of Cybernetics, TalTech, Ehitajate tee 5, 19086 Tallinn, Estonia

[3]Faculty of Technology, Research Unit of Sustainable Chemistry, University of Oulu, Pentti Kaiteran katu 1, 90014 Oulu, Finland

[4]Nanostructured Materials and Interfaces, Zernike Institute for Advanced Materials, University of Groningen, Nijenborgh 4, 9747AG Groningen, The Netherlands

[5]Soft Condensed Matter, Debye Institute for Nanomaterials Science, Utrecht University, Princetonplein 5, 3584 CC Utrecht, The Netherlands

[6]Institute of Molecular and Cell Biology, University of Tartu, Riia 23, 51010 Tartu, Estonia

[7]College of Science, University of Lincoln. Brayford Pool, Lincoln, Lincolnshire. LN6 7TS

[8]Department of Materials Science and Engineering, University of Delaware, Newark, DE 19716, United States

*Corresponding author: Svetlana.Polivtseva@taltech.ee; cvpolcv@gmail.com

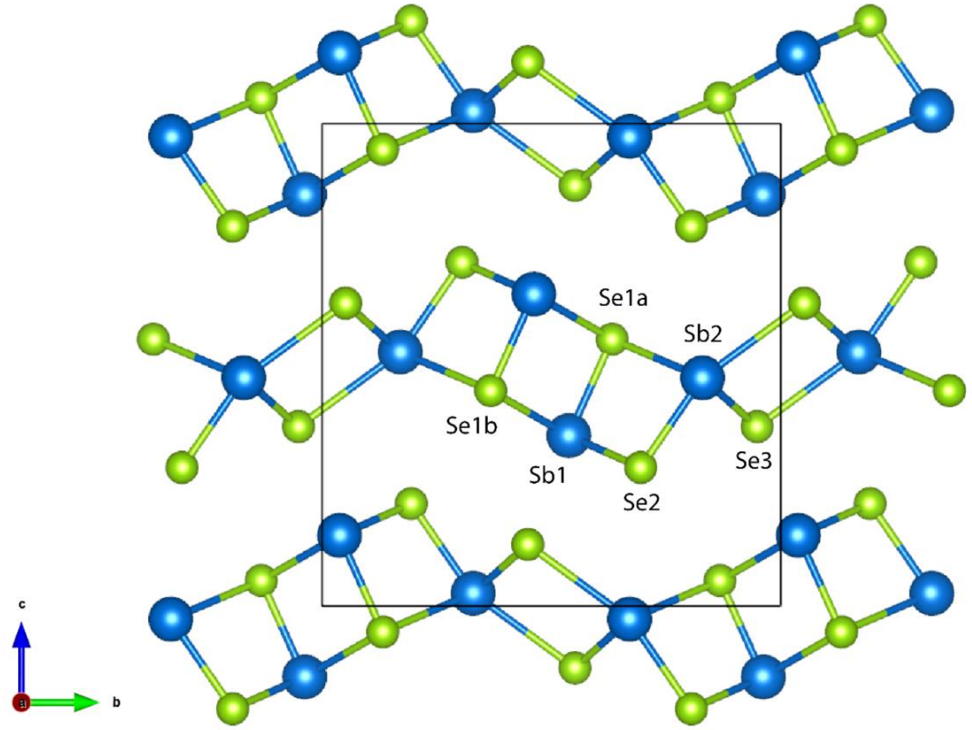

Figure S1 | Schematic illustration of defect-free Sb_2Se_3 structure with the indication of various defect sites considered in this study. Black lines outline the orthorhombic unit cell. Blue and green spheres represent Sb and Se atoms, respectively. There are two non-equivalent Sb sites designated Sb1 and Sb2 and three non-equivalent Se sites designated Se1, Se2, and Se3. Se1a and Se1b are both Se1 types of positions but can form different dimers in combination with the Sb1 position.

Table S1 | Results of the DFT calculations performed for Sb_2Se_3 bulk and defects therein. The following defects were considered: Sb and Se vacancies, Ag substitutional atoms on Sb sites, and combinations of Ag substitutional atoms with adjacent Se vacancy (dimers). The energy difference ΔE is only considered within a composition and is defined with respect to the lowest-energy defect (marked in boldface) found for the particular composition. The electronic ground state (EGS) of several low-energy configurations is given as M (metal), SM (semi-metal), or SC (semiconductor). Electronic bandgap values are also given for semiconductors.

Type	Defect	Composition	ΔE (eV/atom)	EGS	Bandgap (eV)
Bulk	Defect-free Sb_2Se_3	$Sb_{24}Se_{36}$	**0.000**	SC	0.77
Sb vac.	V_{Sb1}	$Sb_{23}Se_{36}$	0.074	M	N.A.
	V_{Sb2}	$Sb_{23}Se_{36}$	**0.000**	M	N.A.
Se vac.	V_{Se1}	$Sb_{24}Se_{35}$	0.029	SC	0.64
	V_{Se2}	$Sb_{24}Se_{35}$	0.036	SC	0.67
	V_{Se3}	$Sb_{24}Se_{35}$	**0.000**	SC	0.65
Ag_{sub}	Ag_{Sb1}	$Ag_1Sb_{23}Se_{36}$	0.322		
	Ag_{Sb2}	$Ag_1Sb_{23}Se_{36}$	**0.000**	M	N.A.
$Ag_{sub}V_{Se}$	$Ag_{Sb1}V_{Se1a}$	$Ag_1Sb_{23}Se_{35}$	0.030	SC	0.48
	$Ag_{Sb1}V_{Se1b}$	$Ag_1Sb_{23}Se_{35}$	0.349		
	$Ag_{Sb1}V_{Se2}$	$Ag_1Sb_{23}Se_{35}$	**0.000**	SC	0.74
	$Ag_{Sb2}V_{Se1}$	$Ag_1Sb_{23}Se_{35}$	0.463		
	$Ag_{Sb2}V_{Se2}$	$Ag_1Sb_{23}Se_{35}$	0.093	SC	0.66
	$Ag_{Sb2}V_{Se3}$	$Ag_1Sb_{23}Se_{35}$	0.298		

Table S2 | Solution compositions used to introduce Ag(I) dopants into Sb_2Se_3. The activated Sb_2Se_3 films were treated in solutions for 90 min at ~210 °C.

Treatment		Auxiliary chemicals				
		Glycerol	$AgNO_3$ M (mM)	$NaHCO_3$ M (mM)	NaCl M (mM)	$SbCl_3$ M (mM)
Activation	pristine	+				
Doping	Ag: $NaHCO_3$	+	0.224	2.17		
Cation exchange	$AgNO_3$	+	0.224			
	Ag: NaCl	+	0.224		2.17	
	Ag: $SbCl_3$: NaCl	+	0.224		2.17	44

Parent solutions

$AgNO_3$ solution: 37 mg of $AgNO_3$ is added to 100 ml of glycerol. The solution is then heated on a hot plate to 60-70 °C for complete dissolution for about 20 min. Then, a clear solution is cooled down and stored in the fridge conditions.

$NaHCO_3$ / NaCl solutions: 18 mg of $NaHCO_3$ or 12.6 mg NaCl is dissolved in 100 ml of glycerol at around 60-70 °C for one h. Then, a clear solution is cooled down and stored in ambient conditions.

$SbCl_3$ solution: 4.0 g of $SbCl_3$ is dissolved in 20 ml of methanol at room temperature.

Cation exchange solutions

10 ml Ag: $NaHCO_3$: 1 ml of the parent $NaHCO_3$ solution is added to 5 ml of glycerol and warmed to 80 °C for 5 min (S1). Then, 1 ml of the parent $AgNO_3$ solution and 3 ml of fresh glycerol are added to the S1 and mixed for about 1-2 min to create a clear solution.

10 ml $AgNO_3$: 1 ml of the parent $AgNO_3$ solution is added to 9 ml of glycerol and warmed to 80 °C for 5 min.

10 ml Ag: NaCl: 1 ml of the parent NaCl solution is added to 5 ml of glycerol and warmed to 80 °C for 5 min (S2). Then, 1 ml of the parent $AgNO_3$ solution and 3 ml of fresh glycerol are added to the S2 and mixed for about 1-2 min to create a clear solution.

10 ml Ag: $SbCl_3$: NaCl: 0.5 ml of the parent $SbCl_3$ solution is added to 5 ml of glycerol (S3), mixed, and left on a hot plate at 100 °C until the evaporation of methanol is completed (around 7-10 min). Then, 1 ml of the parent $AgNO_3$ solution, 1 ml of the parent NaCl solution, and 3 ml of fresh glycerol are added to the S3 and mixed for 1-2 min to create a clear solution.

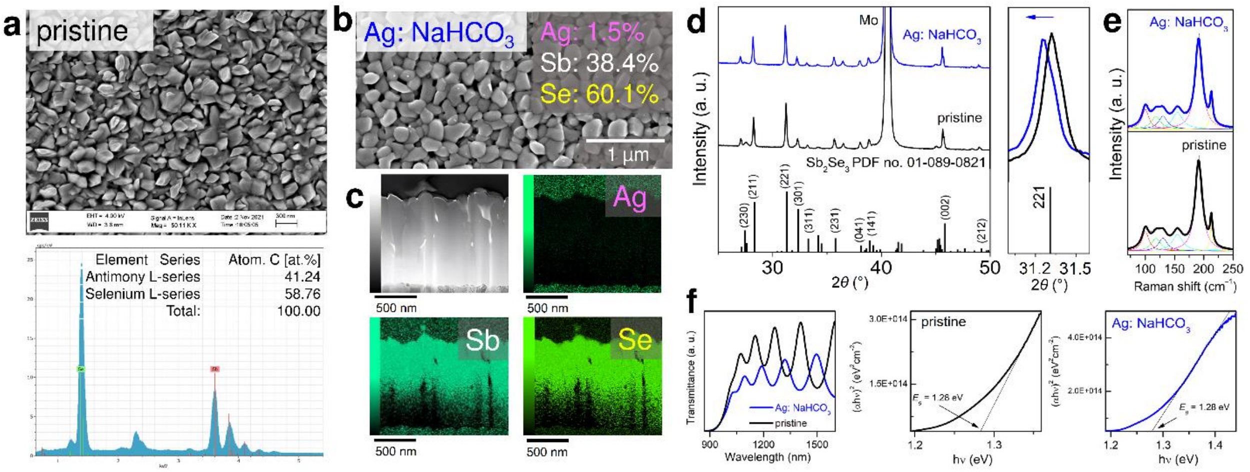

Figure S2 | (**a**) SEM/EDS analysis of the pristine Sb_2Se_3 films inverted from SnSe matrices and activated in glycerol for 30 min at ~210 °C. (**b**) SEM images and TEM/EDS analysis of Ag-doped Sb_2Se_3 films treated in solutions containing $AgNO_3$ and $NaHCO_3$ denoted as Ag: $NaHCO_3$. (**c**) Cross-sectional STEM images with corresponding EDS elemental mappings for Ag-doped Sb_2Se_3 sample derived from Ag: $NaHCO_3$ solution. (**d**) XRD patterns, (**e**) Raman spectra, and (**f**) UV-Vis data for pristine Sb_2Se_3 films and Ag-doped Sb_2Se_3 samples derived from Ag: $NaHCO_3$ solution. Structural, compositional, and optical characteristics were averaged using measurement data from three independent repeats.

Photoluminescence spectroscopy data

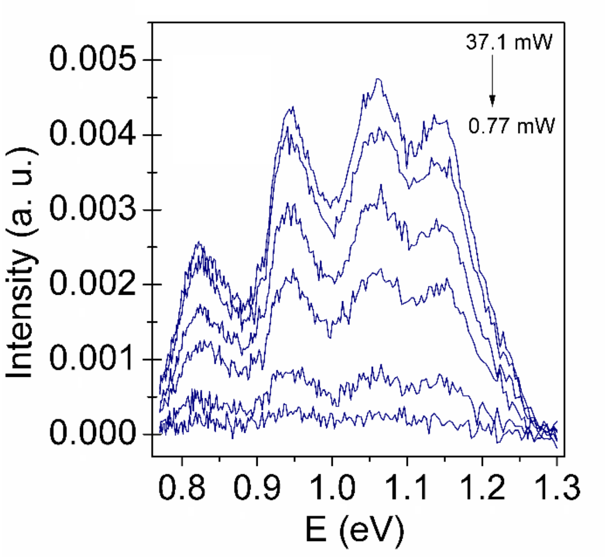

Figure S3 | Excitation power dependence of PL spectra for Ag-doped Sb_2Se_3 sample derived from Ag: $NaHCO_3$ solutions.

The thermal activation energies for all bands can be obtained from the Arrhenius plot (See Figure 1c in the main text), where the dependence of ln(Φ) versus 1000/T was fitted by using the theoretical expression for discrete energy levels:

$$\Phi(T) = \Phi_0/\left(1 + \alpha_1 T^{3/2} + \alpha_2 T^{3/2} exp(-E_A/kT)\right) \qquad \text{(S1)}$$

where Φ is integrated intensity, α_1, and α_2 are the process rate parameters, and E_A is the thermal activation energy.

The emission energy from a donor-acceptor pair separated by a distance r can be calculated using the following equation:

$$h\nu_{max} = E_g - (E_a + E_d) + \frac{e^2}{4\pi\varepsilon_0\varepsilon r} \qquad \text{(S2)}$$

where E_g is the bandgap energy, E_a and E_d are the acceptor and donor ionization energies, respectively, r is the distance between the donor and acceptor, e is the electron charge, ε is the static dielectric constant, and ε_0 is the permittivity of vacuum. The last term of Eq. (S2) describes the Coulomb interaction between the donor and acceptor defects.

Table S3 | Atomic percentages of silver, antimony, and selenium according to the SEM-EDX data. The elemental composition of Ag-doped samples is presented according to the TEM-EDX data. Structural parameters such as the main crystalline phase, the average crystallite size (D), and lattice parameters are determined using XRD patterns. Each parameter was determined by averaging the values obtained from three measurements for each sample. The error represents the standard deviation.

Treatment		elements (at. %)			main phase	D (nm) (± 1)	Lattice parameters (Å) (± 0.001)		
		Sb	Se	Ag			*a*	*b*	*c*
Activation	pristine	41.2	58.8	0.0	**Sb_2Se_3**	79	11.791	3.975	11.625
Doping	Ag: $NaHCO_3$	38.4	60.1	1.5	**Sb_2Se_3**	84	11.782	3.973	11.647
Cation exchange	$AgNO_3$	35.2	54.7	10.1	Sb_2Se_3	85	11.778	3.972	11.609
					$AgSbSe_2$	48	5.785		
	Ag: NaCl	36.5	49.0	14.5	Sb_2Se_3	87	11.784	3.972	11.610
					**$AgSbSe_2$**	64	5.782		
	Ag: $SbCl_3$: NaCl	29.5	50.3	20.2	Sb_2Se_3	93	11.785	3.970	11.621
					**$AgSbSe_2$**	67	5.780		
Reference	PDF Card No.: 01-071-9229				$AgSbSe_2$	Space group: 225 Fm-3m			
	PDF Card No.: 01-089-0821				Sb_2Se_3	Space group: 62 Pbnm			

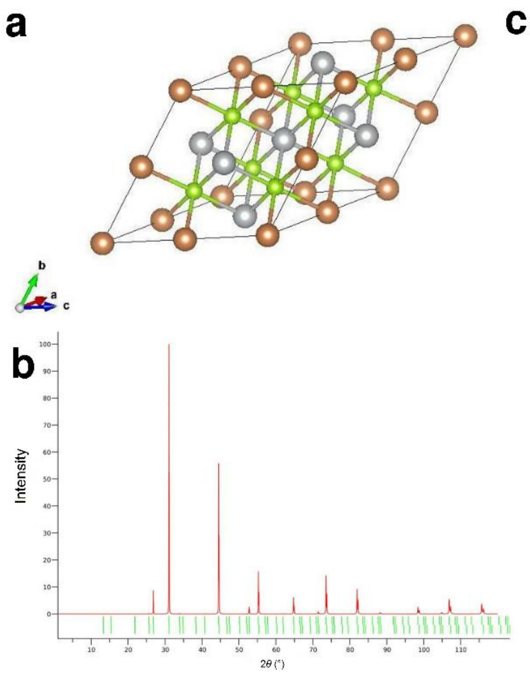

c

IR	deg	THz	cm^-1	
T1u		-0.0954878428	-3.18509248443	
T2u		1.1468292570	38.25363669649	
T1u		1.4465398730	48.25078400378	
Eu		1.5776118893	52.62282217949	
A2u		1.9638899721	65.50751390936	
T1u		2.1744583079	72.53123131831	
T2u		2.2724611693	75.80021476317	
T1u		2.2802542795	76.06016174700	
Eu		2.3803397642	79.39861317465	
T1u		2.8135840873	93.84991081597	
T2u		2.8794248579	96.04609556011	
A2u		3.2704566444	109.0893518306	
Eu		3.2718760341	109.1366969934	
T2g	3	3.2739062266	109.2044160944	active
T1g	3	3.4674308557	115.6596236227	nonactive
Eg	2	3.6600559004	122.0848246137	active
A1g	1	4.4354400732	147.9485390816	active
T2g	3	4.4634093221	148.8814813479	active
A2u	1	4.8217953549	160.8358058580	
T1u		4.9006304107	163.4654279793	

Figure S4 | (**a**) Rock-salt type structure of $AgSbSe_2$ (Fd-3m space group) with disordered Ag/Sb atoms. Grey, brown, and green spheres represent Ag, Sb, and Se atoms. (**b**) Simulated XRD data of rock-salt $AgSbSe_2$. (**c**) The phonon's frequencies are calculated at the Γ-point of the Brillouin zone, q-point (0, 0, 0).

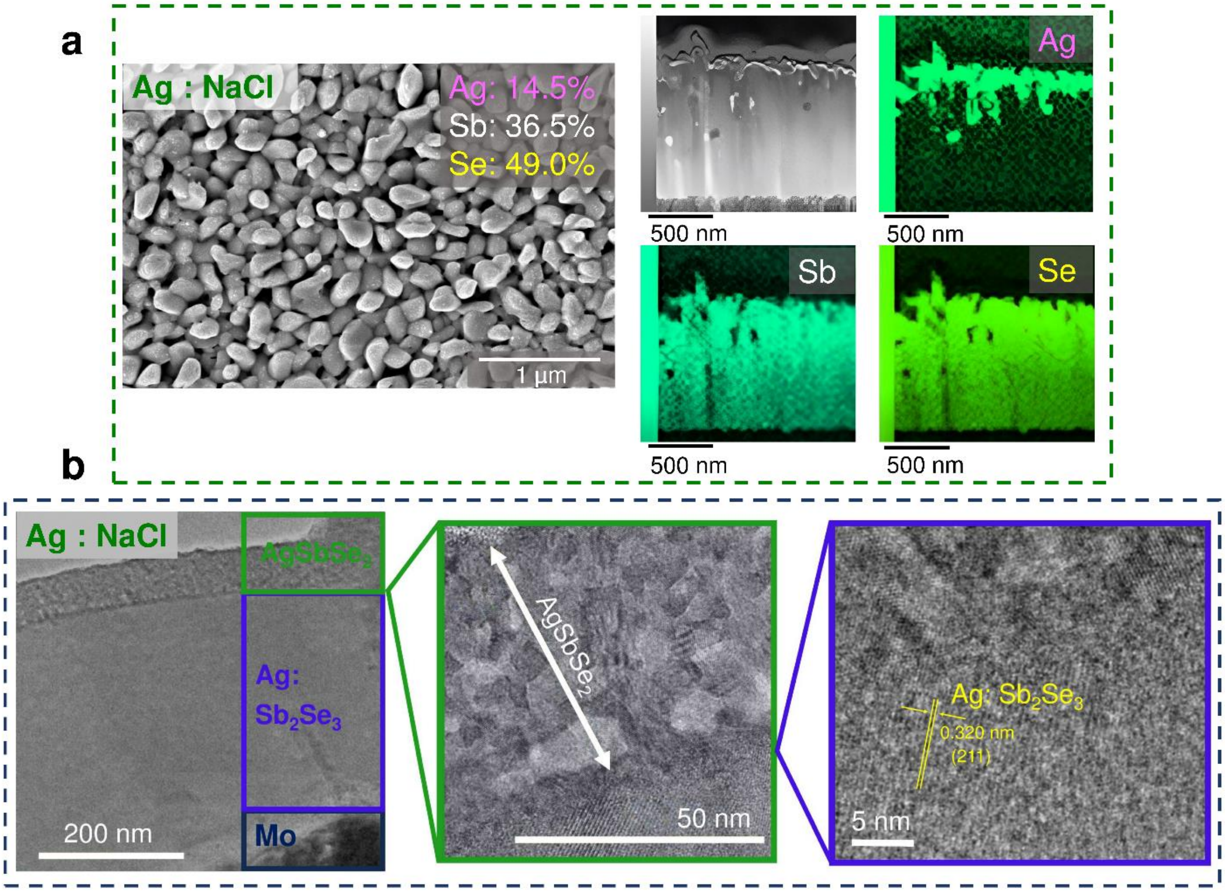

Figure S5 | (**a**) SEM image, TEM/EDS analysis, and cross-sectional STEM images with corresponding EDS elemental mappings and (**b**) cross-sectional TEM and HR-TEM images of the Ag: Sb_2Se_3/$AgSbSe_2$ interface for Ag: Sb_2Se_3 sample derived from Ag: NaCl solution. Compositional characteristics were averaged using measurement data from three independent repeats.

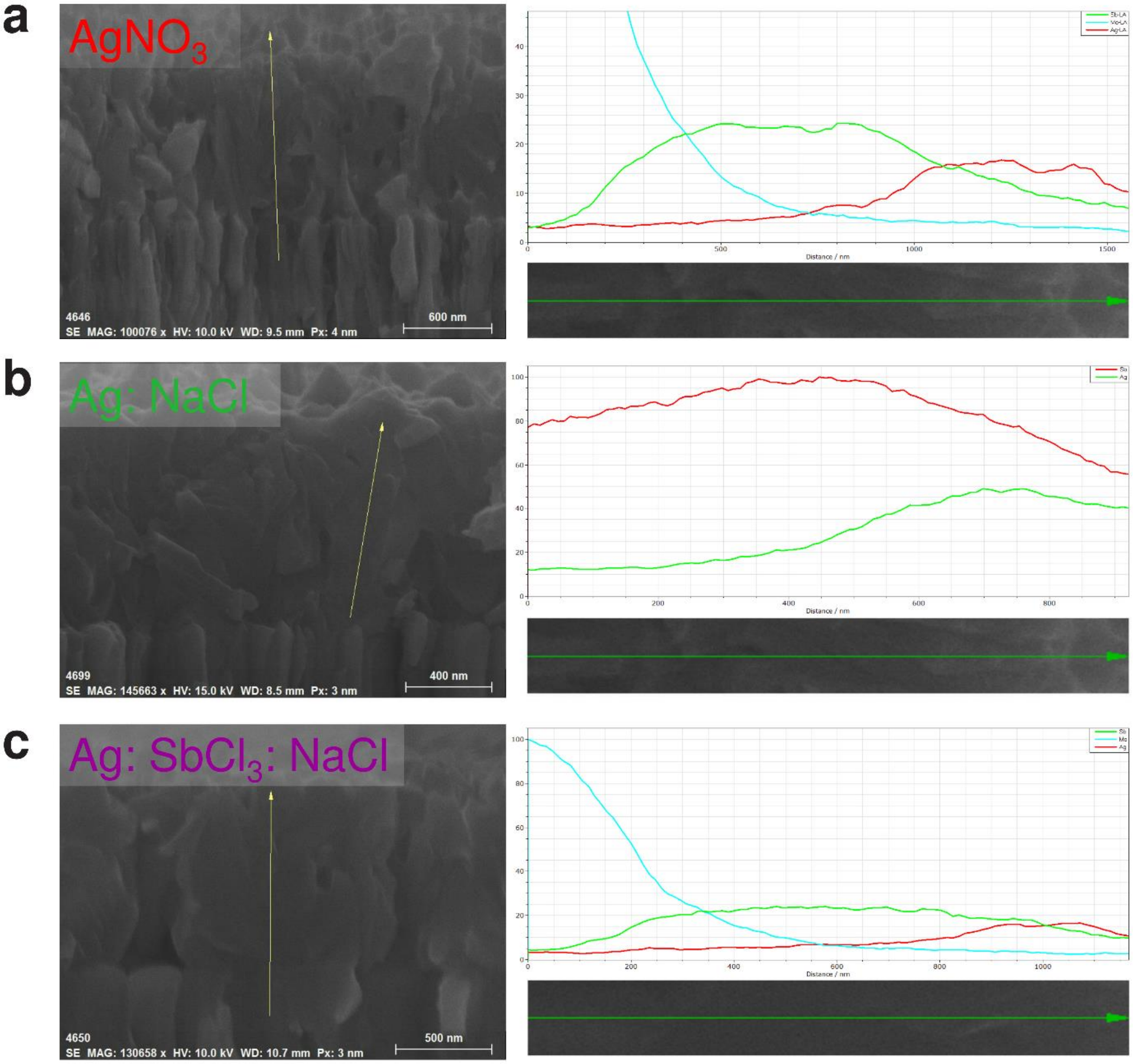

Figure S6 | EDX line scans of Sb and Ag in the inverted Ag: Sb_2Se_3 films depending on the applied solution system: (**a**) $AgNO_3$, (**b**) Ag: NaCl, and (**c**) Ag: $SbCl_3$: NaCl. A gradient decrease of Ag and an increase of Sb along the thickness of the films is observed.

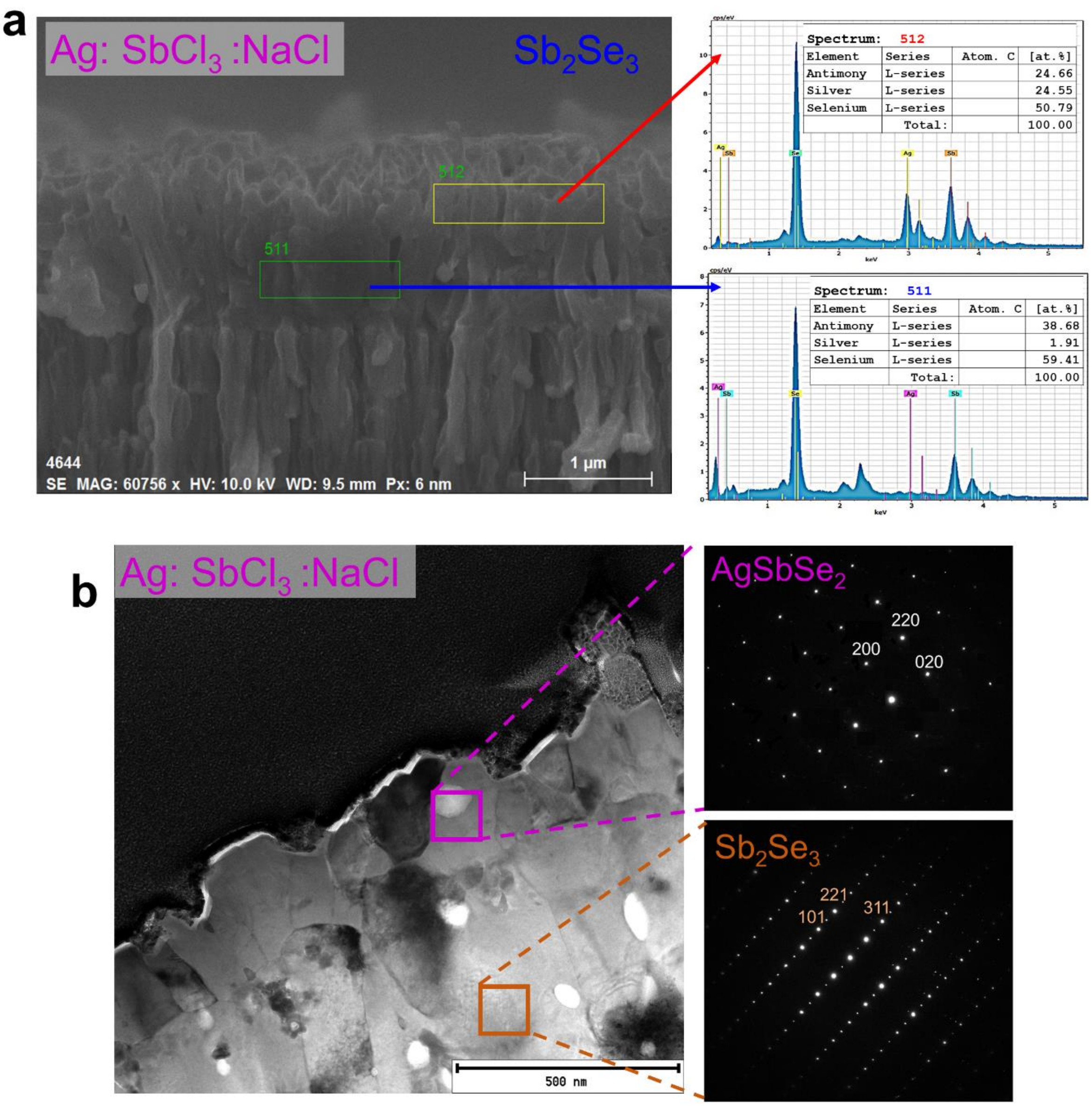

Figure S7 | (a) Cross-sectional SEM-EDX data on silver, antimony, and selenium contents recorded for a two-layered sample derived from Ag: $SbCl_3$: NaCl solution. According to EDX data, the top layer is idealized to $AgSbSe_2$ composition, while the bottom layer has an approximate composition of $Ag_2Sb_{38}Se_{60}$ idealized to $Ag_{0.1}Sb_{1.9}Se_3$. (**b**) Cross-sectional image of a two-layered $AgSbSe_2$/Sb_2Se_3 sample with SAED patterns. Compositional characteristics were averaged using measurement data from three independent repeats.

Defect-driven transformation applied to Sb_2S_3 thin film matrices.

$Sb_2Se_3 + Ag^+ \rightarrow AgSbSe_2 + Sb^{3+}$ ($\Delta G^{200°C} \approx -8.5$ kcal/mol) (S3)

$Sb_2S_3 + Ag^+ \rightarrow AgSbS_2 + Sb^{3+}$ ($\Delta G^{200°C} \approx +17.5$ kcal/mol) (S4)

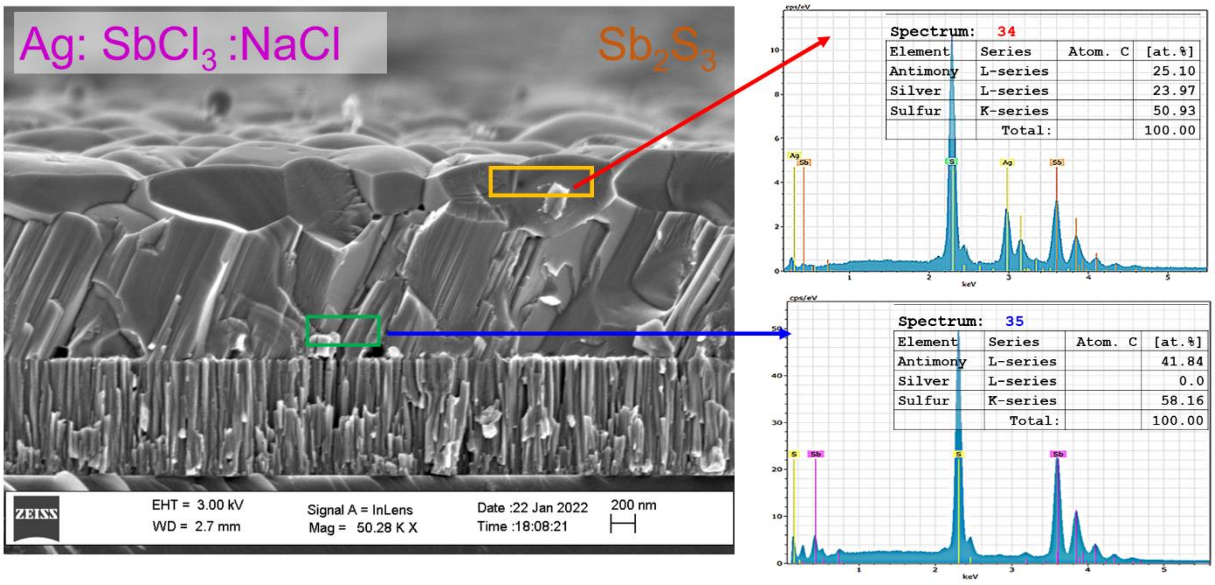

Figure S8 | Cross-sectional SEM-EDX data on silver, antimony, and sulfur contents recorded for a two-layered sample derived from Ag: $SbCl_3$: NaCl solution. According to EDX data, the top layer has a composition idealized as $AgSbS_2$, while the bottom layer close to a substrate has an approximate composition idealized to $Sb_{2.1}S_{2.9}$. Compositional characteristics were averaged using measurement data from three independent repeats.

XPS data

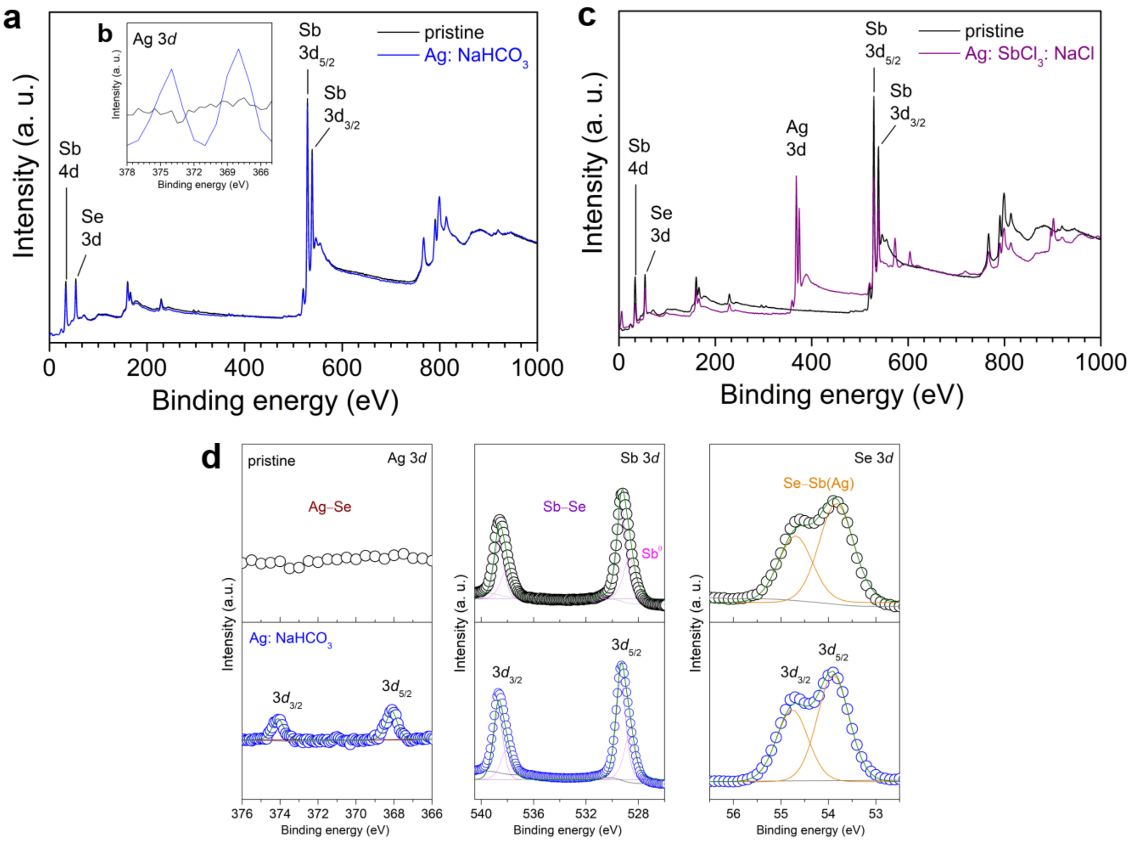

Figure S9 | X-ray photoelectron spectroscopy showing (**a**) XPS survey spectra and (**b**) the Ag 3*d* core level peak region of the pristine Sb_2Se_3 film and Ag-doped Sb_2Se_3 sample treated in Ag: $NaHCO_3$ solutions. (**c**) Comparison of XPS survey spectra of the pristine Sb_2Se_3 film and the Ag: Sb_2Se_3 sample derived from Ag: $SbCl_3$: NaCl solutions. (**d**) Comparison of high-resolution spectra of Ag 3*d*, Sb 3*d*, and Se 3*d* core level peak regions.

Table S4 | Quantification XPS data of the pristine Sb_2Se_3 and Ag: Sb_2Se_3 samples derived from Ag: $NaHCO_3$ and Ag: $SbCl_3$: NaCl solutions.

Treatment	Raw Peak Area (cps eV)		
	Ag 3*d*	Sb 3*d*	Se 3*d*
pristine	0	282090.9	17625.3
Ag: $NaHCO_3$	3391.3	256517.5	17074.8
Ag: $SbCl_3$: NaCl	142606.8	154011.1	16479.1

Table S5 | ICP-MS data for Ag contents accumulated in the Ag: Sb_2Se_3 films with respect to Ag concentrations fixed in solutions. The values are averaged from three repeats.

Sample type	Sample Id	Ag 107 (mg/L)	Mean Ag value (mg/L)	Yield (%)
Solutions	SGly-1	0.000		
	SGly-2	0.000	0.000	
	SGly-3	0.000		
	Sgly-$AgNO_3$-1	23.149		
	Sgly-$AgNO_3$-2	23.789	23.272	
	Sgly-$AgNO_3$-3	22.877		
Layers	F$AgNO_3$-1	10.948		47.04
	F$AgNO_3$-2	11.452	11.251	49.21
	F$AgNO_3$-3	11.353		48.78
	FAg-$NaHCO_3$-1	0.258		1.11
	FAg-$NaHCO_3$-2	0.474	0.348	2.04
	FAg-$NaHCO_3$-3	0.312		1.34
	FAg-NaCl-1	17.361		74.60
	FAg-NaCl-2	16.694	17.024	71.73
	FAg-NaCl-3	17.019		73.13
	FAg-NaCl-SbCl-1	21.475		92.28
	FAg-NaCl-SbCl-2	20.648	21.063	88.72
	FAg-NaCl-SbCl-3	21.066		90.52

Solution characterization

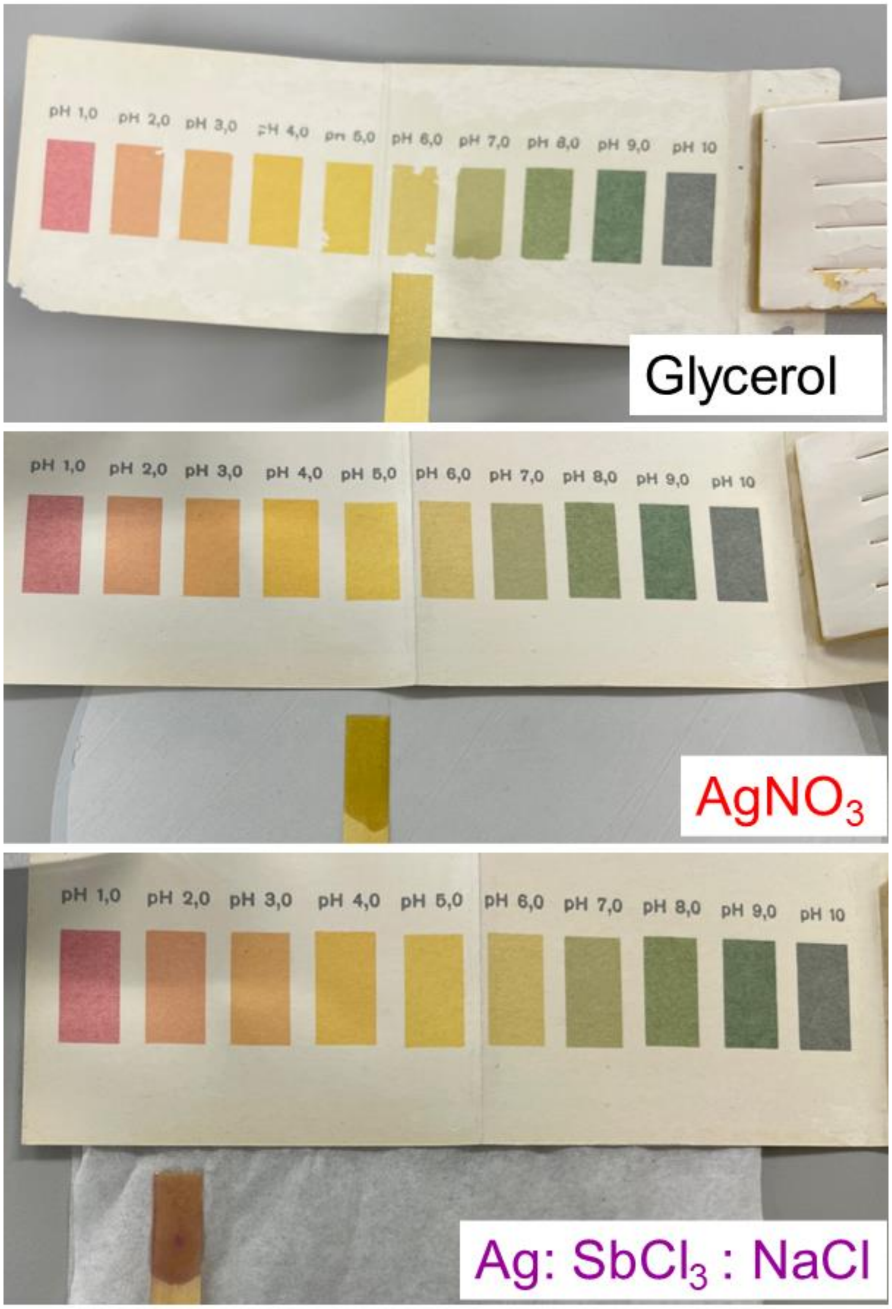

Figure S10 | pH units recorded for pure glycerol and solutions of $AgNO_3$ and Ag: $SbCl_3$: NaCl used for cation exchange.

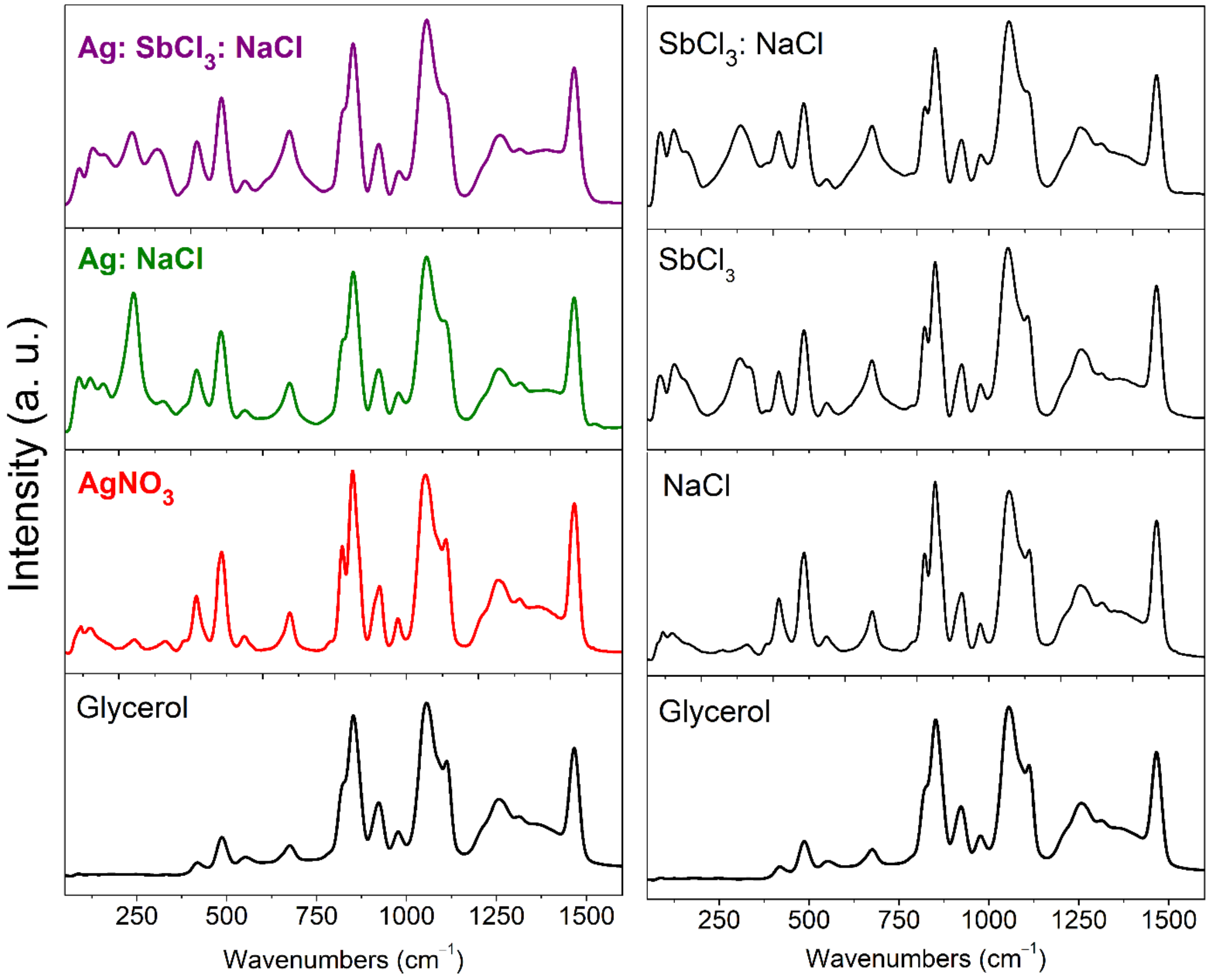

Figure S11 | Raman spectra of pure glycerol and solutions containing $AgNO_3$, NaCl, $SbCl_3$, and mixtures.

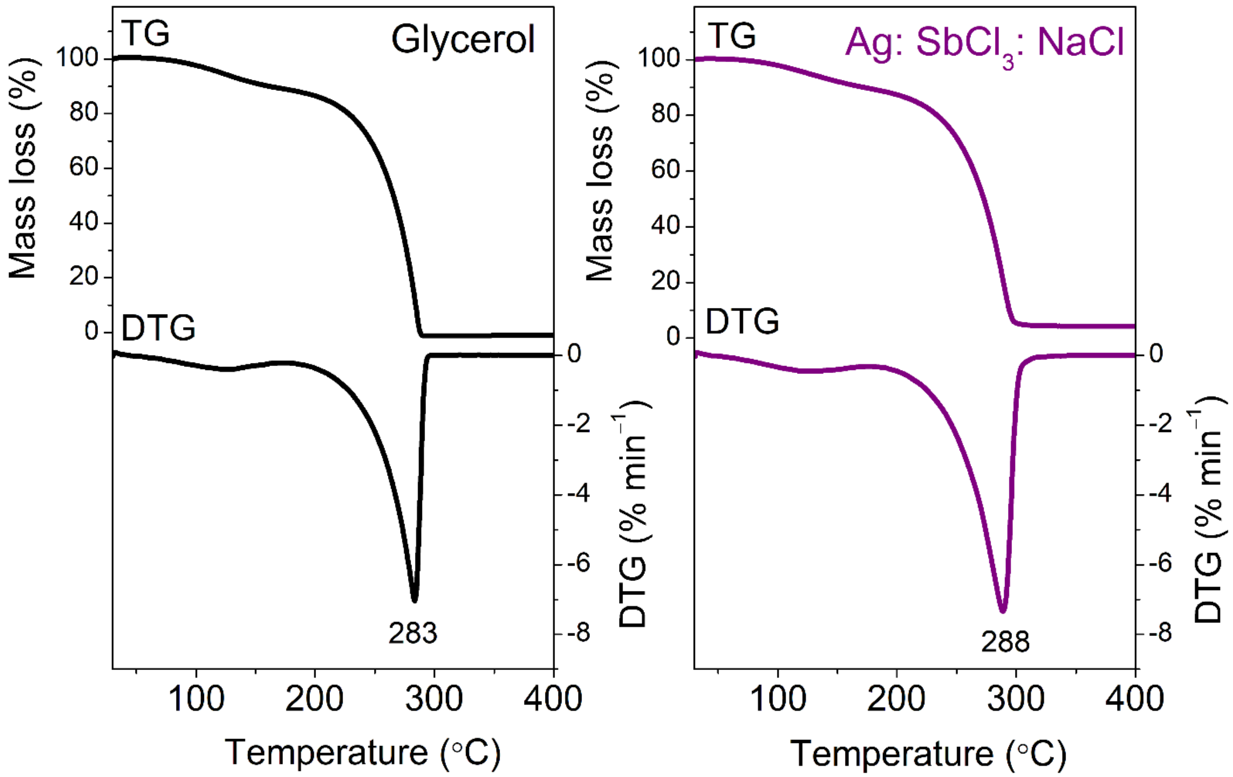

Figure S12 | TG and DTG curves of pure glycerol and Ag: $SbCl_3$: NaCl solution measured by the TG/EGA-MS system in flowing argon with an Ar flow rate of 60 mL min^{-1} and heating rate of 10 °C min^{-1}.

Table S6 | Computational study of complex compounds formed in glycerol solutions containing $AgNO_3$, NaCl, and $SbCl_3$

**Atoms**	Energy of isolated atoms [Ha]
C	-37,554
H	-0,5
O	-74,592432
Sb	-194,6147
Cl	-459,3193047
Ag	-146,0840473
Na	-161,7926326

Ha=Hartree=27.211386 eV

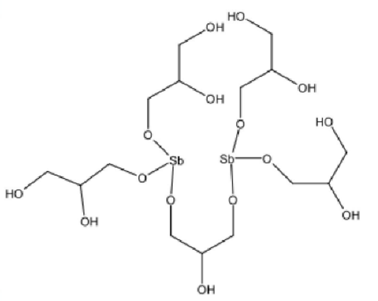

	Number of specific Atoms	*Total energy E [Ha]*	*Total number of atoms (N)*	*Energy per atom E/N [Ha]*	*DIFF/N [Ha]*	*DIFF/N [eV]*	*Description*	*Diss. energy / total energy (%)*
**Model 1**		-2187,9658	66	-33,15099697			Calculated energy	
					-1,5081806	-41,039685	Energy of complete dissociation	4,549427601
		-2088,42588	66	-31,64281636			Total energy for isolated atom	
C	15							
H	34							
O	15							
Sb	2							
Cl	0							

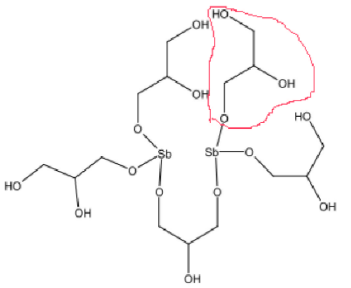

	Number of specific Atoms	*Total energy E [Ha]*	*Total number of atoms (N)*	*Energy per atom E/N [Ha]*	*DIFF/N [Ha]*	*DIFF/N [eV]*	*Description*	*Diss. energy / total energy (%)*
**Model 1 Cl1**		-2305,3443	54	-42,69156111			Calculated energy	
					-1,8062669	-49,1510256	Energy of complete dissociation	4,230969376
		-2207,80589	54	-40,88529423			Total energy for isolated atom	
C	12							
H	27							
O	12							
Sb	2							
Cl	1							

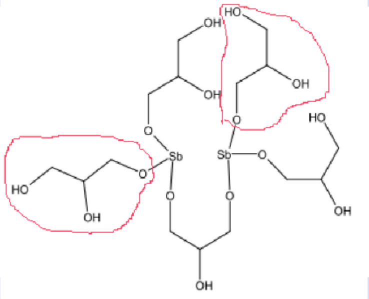

	Number of specific Atoms	*Total energy E [Ha]*	*Total number of atoms (N)*	*Energy per atom E/N [Ha]*	*DIFF/N [Ha]*	*DIFF/N [eV]*	*Description*	*Diss. energy / total energy (%)*
**Model 1 Cl2**		-2422,71801	42	-57,68376214			Calculated energy	
					-2,2745741	-61,8943147	Energy of complete dissociation	3,943179202
		-2327,1859	42	-55,40918803			Total energy for isolated atom	
C	9							
H	20							
O	9							
Sb	2							
Cl	2							

		Number of specific Atoms	Total energy E [Ha]	Total number of atoms (N)	Energy per atom E/N [Ha]	DIFF/N [Ha]	DIFF/N [eV]	Description	Diss. energy / total energy (%)
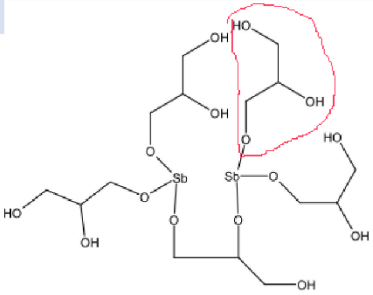	**Model 2 Cl1**		-2305,33349	54	-42,69136093			Calculated energy	
						-1,8060667	-49,1455783	Energy of complete dissociation	4,230520303
			-2207,80589	54	-40,88529423			Total energy for isolated atom	
	C	12							
	H	27							
	O	12							
	Sb	2							
	Cl	1							
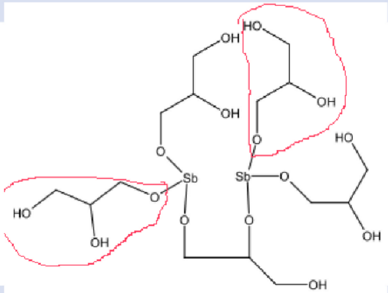	**Model 2 Cl2**		-2422,7254	42	-57,6839381			Calculated energy	
						-2,2747501	-61,8991026	Energy of complete dissociation	3,943472202
			-2327,1859	42	-55,40918803			Total energy for isolated atom	
	C	9							
	H	20							
	O	9							
	Sb	2							
	Cl	2							
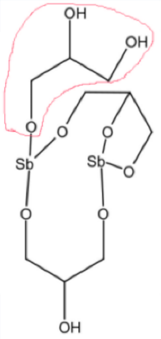	**Model 3 Cl1**		-1620,0112	26	-62,30812308			Calculated energy	
						-3,5801501	-97,420848	Energy of complete dissociation	5,745880235
			-1526,9273	26	-58,72797295			Total energy for isolated atom	
	C	6							
	H	11							
	O	6							
	Sb	2							
	Cl	1							
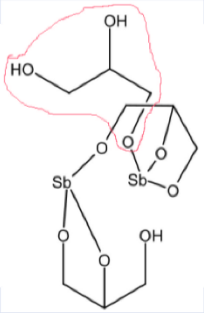	**Model 4 Cl1**		-1619,832	26	-62,30123077			Calculated energy	
						-3,5732578	-97,2332987	Energy of complete dissociation	5,735453019
			-1526,9273	26	-58,72797295			Total energy for isolated atom	
	C	6							
	H	11							
	O	6							
	Sb	2							
	Cl	1							

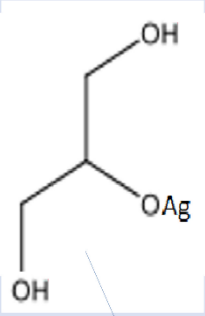

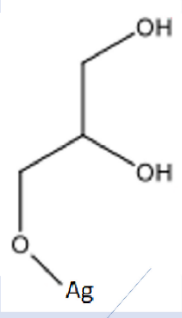

	Number of specific Atoms	Total energy E [Ha]	Total number of atoms (N)	Energy per atom E/N [Ha]	DIFF/N [Ha]	DIFF/N [eV]	Description	Diss. energy / total energy (%)
**Model 1_1 Ag1**		-488,0473	14	-34,86052143			Calculated energy	
**Model 1_2 Ag1**					-0,1445683	-3,93390482	Energy of complete dissociation	0,41470503
		-486,023343	14	-34,71595309			Total energy for isolated atom	
C	3							
H	7							
O	3							
Sb	0							
Ag	1							

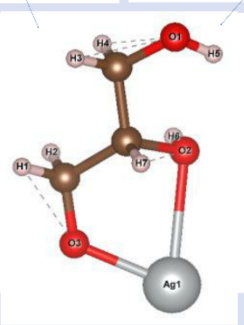

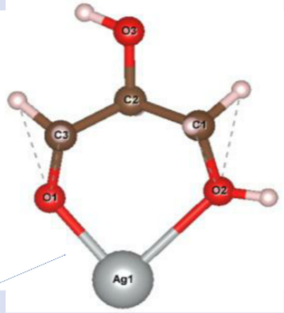

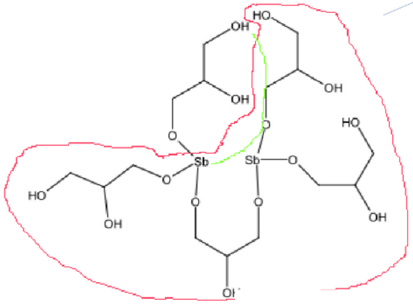

	Number of specific Atoms	Total energy E [Ha]	Total number of atoms (N)	Energy per atom E/N [Ha]	DIFF/N [Ha]	DIFF/N [eV]	Description	Diss. energy / total energy (%)
**Model 1_3 Ag1**		-488,045	14	-34,86035714			Calculated energy	
					-0,1444041	-3,92943438	Energy of complete dissociation	0,414235716
		-486,023343	14	-34,71595309			Total energy for isolated atom	
C	3							
H	7							
O	3							
Sb	0							
Ag	1							

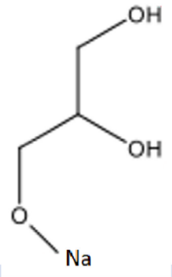

	Number of specific Atoms	Total energy E [Ha]	Total number of atoms (N)	Energy per atom E/N [Ha]	DIFF/N [Ha]	DIFF/N [eV]	Description	Diss. energy / total energy (%)
**Model 1_1 Na1**		-503,798382	14	-35,98559871			Calculated energy	
					-0,1476038	-4,0165044	Energy of complete dissociation	0,41017468
		-501,731929	14	-35,8379949			Total energy for isolated atom	
C	3							
H	7							
O	3							
Sb	0							
Na	1							

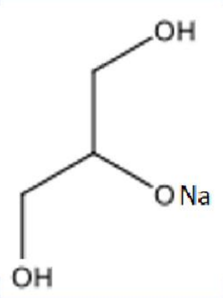

	Number of specific Atoms	Total energy E [Ha]	Total number of atoms (N)	Energy per atom E/N [Ha]	DIFF/N [Ha]	DIFF/N [eV]	Description	Diss. energy / total energy (%)
**Model 1_2 Na1**		-503,799788	14	-35,98569914			Calculated energy	
					-0,1477042	-4,01923701	Energy of complete dissociation	0,410452594
		-501,731929	14	-35,8379949			Total energy for isolated atom	
C	3							
H	7							
O	3							
Sb	0							
Na	1							

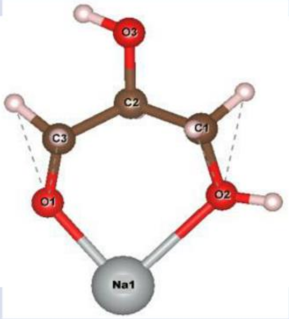

	Number of specific Atoms	Total energy E [Ha]	Total number of atoms (N)	Energy per atom E/N [Ha]	DIFF/N [Ha]	DIFF/N [eV]	Description	Diss. energy / total energy (%)
**Model 1_3 Na1**		-503,802732	14	-35,98590941			Calculated energy	
					-0,1479145	-4,02495882	Energy of complete dissociation	0,411034516
		-501,731929	14	-35,8379949			Total energy for isolated atom	
C	3							
H	7							
O	3							
Sb	0							
Na	1							

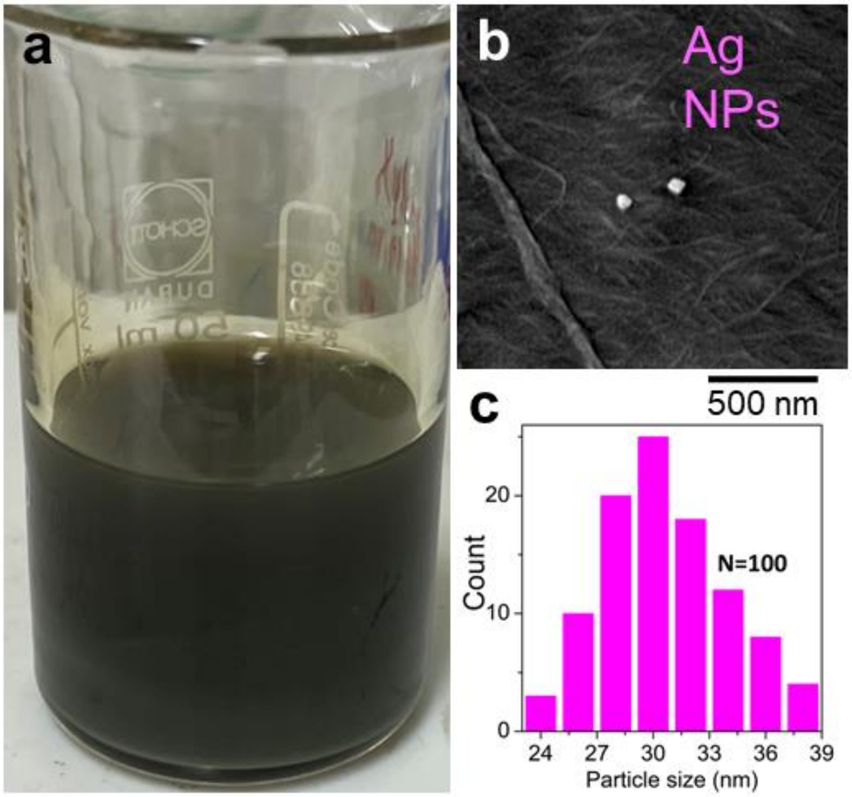

Figure S13 | (**a**) Photo of the solution containing $AgNO_3$ and $NaHCO_3$ at working concentrations specified in Table S2. (**b**) SEM image of Ag NPs attached to a paper filter. Ag NPs are formed in Ag: $NaHCO_3$ solutions upon heating for 5-7 min. (**c**) Histogram of the particle-size distribution.

Fabrication setup for $AgSbSe_2$ samples for antibacterial tests.

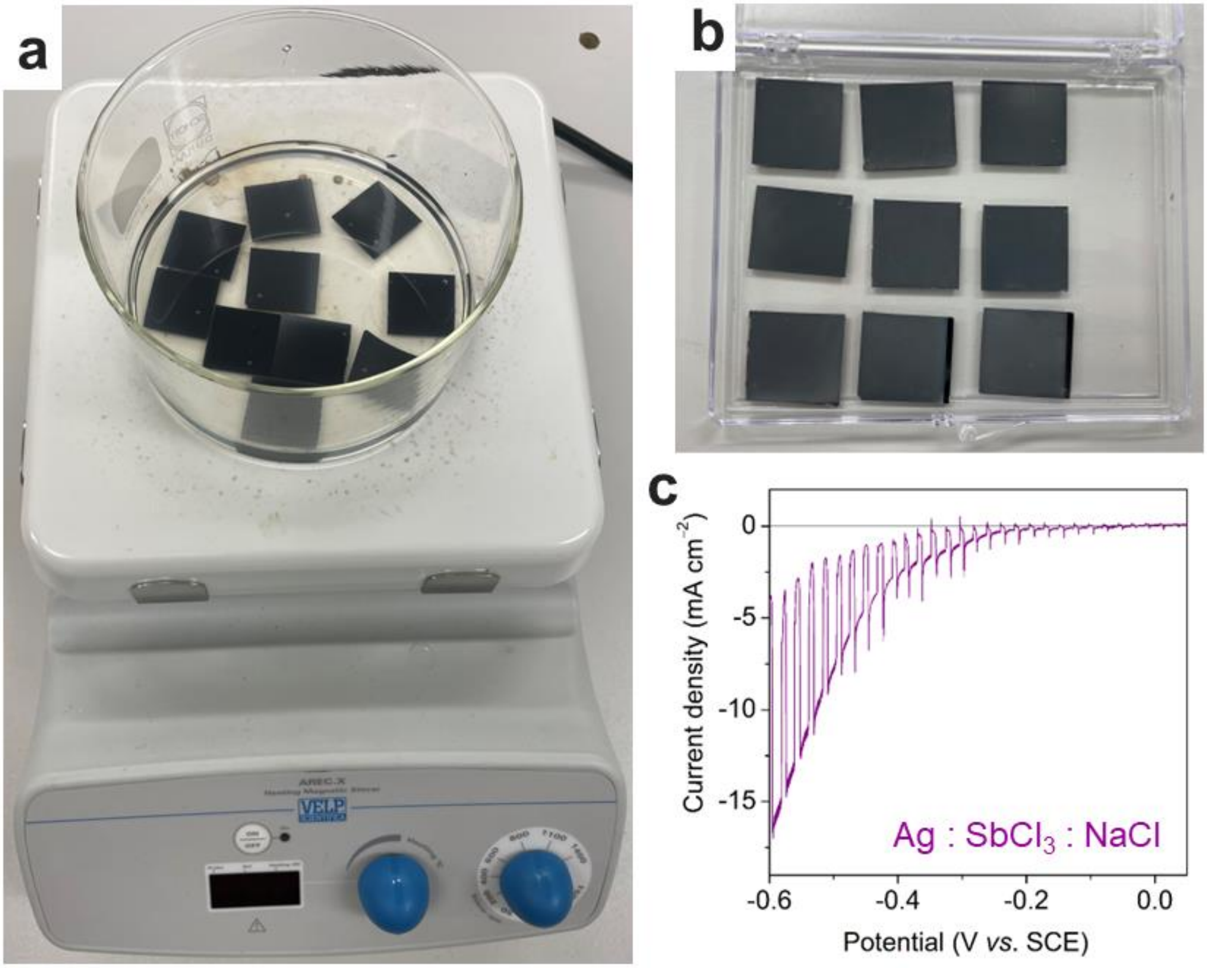

Figure S14 | (**a**) A fabrication setup, (**b**) photo, and (**c**) photoelectrochemical characteristics of resulting Ag: Sb_2Se_3 structures derived from Ag: $SbCl_3$: NaCl solution. The photoelectrochemical response was estimated using a potentiostat (Gamry Reference 3000) in a three-electrode configuration. A platinum wire and saturated calomel electrode were used as the counter and reference electrodes. A white LED lamp was used as a light source, and its intensity was around 30 mW cm^{-2}. Linear sweep voltammograms were measured at a scan rate of 20 mV s^{-1} from negative to positive at room temperature in 1M H_2SO_4 (pH below 1) aqueous solution.

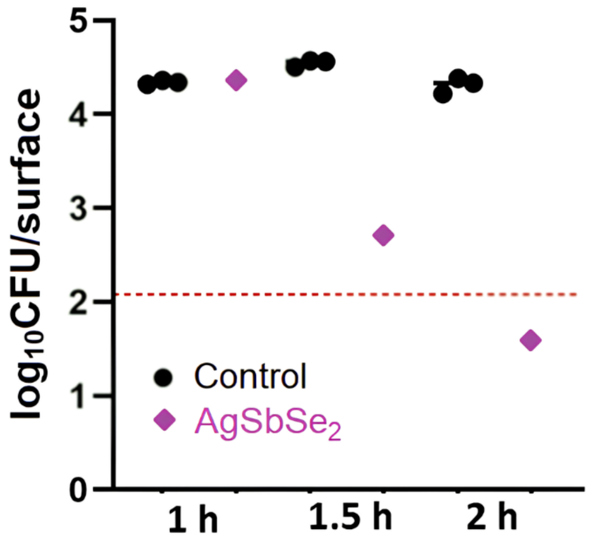

Figure S15 | Antibacterial activity of $AgSbSe_2$ thin film layers derived from Ag: $SbCl_3$: NaCl solutions towards *Escherichia coli* after 1.0, 1.5, and 2.0 h of exposure under visible light illumination. Bacterial numbers are shown as log10 colony-forming units (CFU) on $AgSbSe_2$ thin film layers and neutral control surfaces (borosilicate glass). The red dotted line indicates a detection limit for the assay.

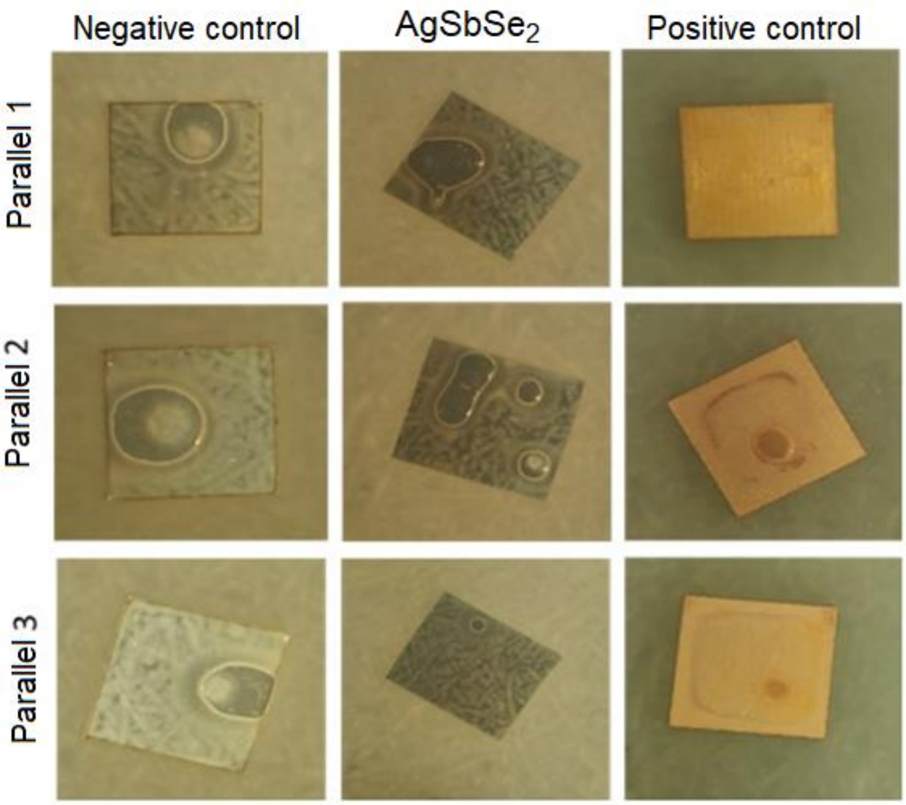

Figure S16 | $AgSbSe_2$ material, negative control samples (stainless steel), and positive control samples (copper) were incubated on a "lawn" of *E.coli* ATCC 8739 for 24 h. Three parallels were used for each surface type. Pictures were taken from below, and bacterial growth is visible under negative control (left) and $AgSbSe_2$ (middle). No growth was observed underneath positive control samples.

$AgSbSe_2$ surfaces were placed onto a Petri dish with LB nutrient agar, to which 10^5 freshly grown cells of E.coli ATCC 8739 were seeded using glass beads to reveal the zone of inhibition derived from metal species realized. The negative control, positive control, and test $ASbSe_2$ samples were then applied onto the active side of the LB agar against freshly seeded bacteria, and the plates were incubated at 37 °C for 24 hours. After 24 h, we detected bacterial growth under $AgSbSe_2$ surfaces and observed no inhibition zone around the samples. Negative control stainless steel surfaces with a presumably neutral effect on bacterial growth show bacterial growth underneath and no inhibition zone around. Positive control copper sheet samples show no bacterial growth underneath and no inhibition zones around. The latter suggested sufficient leaching of copper ions from the positive control to stop bacterial growth. Therefore, the negative control-like growth detected under $AgSbSe_2$ surfaces suggested no leaching-driven antibacterial effect occurring on the surface of $AgSbSe_2$.

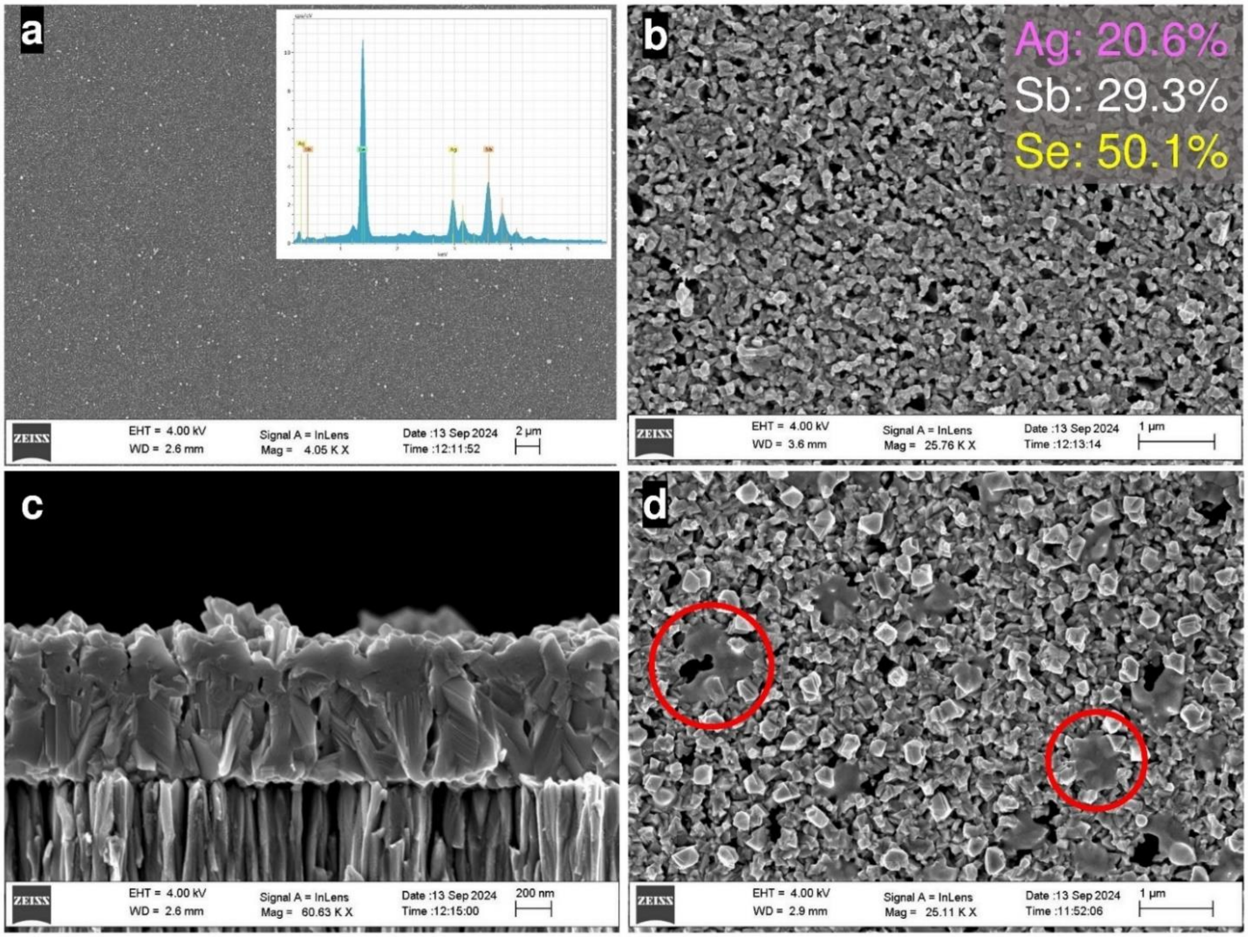

Figure S17 | (**a**, **b**) Top view and (**c**) cross-sectional SEM images with EDS analysis of the samples derived from Ag: $SbCl_3$: NaCl solution and experienced ten cycles of photothermal humidity processing that are as close as possible to conditions used for antibacterial tests. (**d**) Top view SEM image with observed amorphous features (highlighted by red circles). Compositional characteristics were averaged using measurement data from three independent repeats.

According to SEM/EDX data recorded for the samples that experienced testing of photothermal humidity stability, surface features are slightly reorganized at minimal compositional changes (Figure S17 a, b). Cross-sectional images show no voids and visible degradation due to corrosive effects (Figure S17c). Nevertheless, certain areas show the evolution of surface layers towards appearing amorphous features upon partial photo-assisted degradation (Figure S17d). This fact suggests that the potential application of such materials inside the human body or animals may be limited and require separate studies to be concluded. This is also true when such materials have a high surface area-to-volume ratio, e.g., nanoparticle forms.

Table S7 | Photocatalysts used for *Escherichia coli* inactivation.

Sample type	Photocatalysts	Dose (g/L)	Contact time (min)	Efficiency (%)	Light Source	Ref.
Powder/ nanostructure	TiO_2/ CdS	0.1	10	99	VIS	1
	Ag_2WO_4/ g-C_3N_4	4	90	100	VIS	2
	AgBr/ g-C_3N_4	0.1	60	100	VIS	3
	F: ZnO	-	360	99.9	VIS	4
	V:TiO_2/ g-C_3N_4	0.5	60	99.5	VIS	5
	$SnIn_4S_8$/ Bi_2S_3/ Ag QDs	-	240	100	VIS	6
	TiO_2/ $Ag_2C_2O_4$	0.1	30	99.99	VIS	7
	Bi_2MoO_6/ $CuBi_2O_4$	0.8	240	100	VIS	8
	CV/ $Au_{25}(Cys)_{18}$	-	1440	99.99	VIS	9
	ZnO/ Al_2O_3	0.5	240	100	VIS	10
	Cr: TiO_2	0.1	360	70	VIS	11
Thin film on flat substrates	Polyester/ TiON	-	40	100	Sunlight	12
	Ti/ N:TiO_2	-	240	33	UV	13
	Polyester/ TiO_2 / Cu	-	30	100	UVA	14
	Polyester/ CuO_x	-	15	100	Sunlight	15
	PET/ TiO_2/ CuO_x	-	20	100	UVA	16
	Commercial Photocatalytic Windows/ humidity 90%					
	SaniTise™ (Glass/ TiO_2)	-	240	99	UVA	17
	BioClean® (Glass/ TiO_2)	-	240	no activity	UVA	17
	Mo/ Sb_2Se_3/ $AgSbSe_2$	-	120	100	VIS	This work
	Mo/ Sb_2Se_3/ $AgSbSe_2$	-	90	95	VIS	

References:

(1) Gao P.; Liu J.; Zhang T.; Sun D. D.; Ng W. Hierarchical TiO_2/CdS “Spindle-like” Composite with High Photodegradation and Antibacterial Capability under Visible Light Irradiation. *J. Hazard. Mater*. **2012**, 229–230, 209–216.

(2) Li Y.; Li Y.; Ma S.; Wang P.; Hou Q.; Han J.; Zhan S. Efficient Water Disinfection with Ag_2WO_4-doped Mesoporous g-C_3N_4 Under Visible Light. *J. Hazard. Mater*. **2017**, 338, 33–46.

(3) Deng J.; Liang J.; Li M.; Tong M. Enhanced visible-light-driven photocatalytic bacteria disinfection by g-C_3N_4-AgBr. *Colloids Surf. B Biointerfaces.* **2017**, 152, 49–57.

(4) Podporska-Carroll J.; Myles A.; Quilty B.; McCormack D. E.; Fagan R.; Hinder S. J.; Dionysiou D. D.; Pillai S. C. Antibacterial Properties of F-doped ZnO Visible Light Photocatalyst. *J. Hazard. Mater.* **2017**, 324, 39–47.

(5) Shanmugam V.; Sanjeevamuthu S.; Jeyaperumal K. S.; Vairamuthu R. Fabrication of Heterostructured Vanadium Modified g-C_3N_4/TiO_2 Hybrid Photocatalyst for Improved Photocatalytic Performance Under Visible Light Exposure and Antibacterial Activities. *J. Ind. Eng. Chem.* **2019**, 76, 318–332.

(6) Shi H.; Wang C.; Zhao Y.; Liu E.; Fan J.; Ji Z. Highly Efficient Visible Light Driven Photocatalytic Inactivation of E. coli with Ag QDs Decorated Z-scheme Bi_2S_3/$SnIn_4S_8$ Composite. *Appl. Catal. B Environ.* **2019**, 254, 403–413.

(7) Wu X.; Cao L.; Song J.; Si Y.; Yu J.; Ding B. Thorn-like Flexible $Ag_2C_2O_4$/TiO_2 Nanofibers as Hierarchical Heterojunction Photocatalysts for Efficient Visible-Light-Driven Bacteria-Killing. *J. Colloid Interface Sci*. **2020**, 560, 681–689.

(8) Shi H.; Fan J.; Zhao Y.; Hu X.; Zhang X.; Tang Z. Visible Light Driven $CuBi_2O_4$/Bi_2MoO_6 p-n Heterojunction with Enhanced Photocatalytic Inactivation of *E. coli* and Mechanism Insight. *J. Hazard. Mater*. **2020**, 381, 121006.

(9) Hwang, G. B.; Huang, H.; Wu, G.; Shin, J.; Kafizas, A.; Karu, K.; Toit, H. D.; Alotaibi, A. M.; Mohammad-Hadi, L.; Allan, E.; MacRobert, A. J.; Gavriilidis, A.; Parkin, I. P. Photobactericidal Activity Activated by Thiolated Gold Nanoclusters at Low Flux Levels of White Light. *Nat Commun.* **2020**, 11, 1207.

(10) Basu A.; Misra A. J.; Behera M.; Behera S. K.; Nayak A. K.; Dhal N. K.; Mishra A.; Satpathy B. K.; Lundborg C. S.; Tripathy S. K. Photocatalytic Disinfection of Extended-Spectrum Beta-Lactamase Producing Escherichia coli using Alumina/ZnO Heterostructures. *J. Environ. Chem. Eng.* **2021**, 9, 106334.

(11) Gomez-Polo C.; Larumbe S.; Gil A.; Muñoz D.; Fernández L. R.; Barquín L. F.; García-Prieto A.; Fdez-Gubieda M. L.; Muela A. Improved Photocatalytic and Antibacterial Performance of Cr doped TiO_2 Nanoparticles. *Surf. Interfaces.* **2021**, 22, 100867.

(12) Rtimi S.; Pulgarin C.; Bensimon M.; Kiwi J. Evidence for TiON Sputtered Surfaces Showing Accelerated Antibacterial Activity Under Simulated Solar Irradiation. *Sol. Energy* **2013**, 93, 55–62.

(13) Ohtsu N.; Yokoi K.; Saito A. Fabrication of a Visible-Light-Responsive Photocatalytic Antibacterial Coating on Titanium through Anodic Oxidation in a Nitrate/Ethylene Glycol Electrolyte. *Surf. Coat. Technol*. **2015**, 262, 97–102.

(14) Rtimi S.; Ballo M. K. S.; Laub D.; Pulgarin C.; Entenza J. M.; Bizzini A.; Sanjines R.; Kiwi J. Duality in the *Escherichia coli* and Methicillin Resistant Staphylococcus Aureus Reduction Mechanism under Actinic Light on Innovative Co-sputtered Surfaces. *Appl. Catal. A Gen*. **2015**, 498, 185–191.

(15) Suárez L.; Baghriche O.; Rtimi S.; Pulgarin C.; Kiwi J. Sputtered Cu-polyethylene Films Inducing Bacteria Inactivation in the Dark and Under Low Intensity Sunlight. *J. Photochem. Photobiol. A Chem.* **2016**, 330, 163–168.

(16) Rtimi S.; Pulgarin C.; Robyr M.; Aybush A.; Shelaev I.; Gostev F.; Nadtochenko V.; Kiwi J. Insight into the Catalyst/Photocatalyst Microstructure Presenting the Same Composition but Leading to a Variance in Bacterial Reduction under Indoor Visible Light. *Appl. Catal. B Environ.* **2017**, 208, 135–147.

(17) Kisand V.; Visnapuu M.; Rosenberg M.; Danilian D.; Vlassov S.; Kook M.; Lange S.; Pärna R.; Ivask, A. Antimicrobial Activity of Commercial Photocatalytic SaniTise™ Window Glass. *Catalysts* **2022**, 12, 197.
